# Novel MscL agonists that allow multiple antibiotics cytoplasmic access activate the channel through a common binding site

**DOI:** 10.1371/journal.pone.0228153

**Published:** 2020-01-24

**Authors:** Robin Wray, Junmei Wang, Irene Iscla, Paul Blount

**Affiliations:** 1 Department of Physiology, UT Southwestern Medical Center, Dallas, Texas, United States of America; 2 Department of Pharmaceutical Sciences and Computational Chemical Genomics Screening Center, University of Pittsburgh School of Pharmacy, Pittsburg, Pennsylvania, United States of America; Bioinformatics Institute, SINGAPORE

## Abstract

The antibiotic resistance crisis is becoming dire, yet in the past several years few potential antibiotics or adjuvants with novel modes of action have been identified. The bacterial mechanosensitive channel of large conductance, MscL, found in the majority of bacterial species, including pathogens, normally functions as an emergency release valve, sensing membrane tension upon low-osmotic stress and discharging cytoplasmic solutes before cell lysis. Opening the huge ~30Å diameter pore of MscL inappropriately is detrimental to the cell, allowing solutes from and even passage of drugs into to cytoplasm. Thus, MscL is a potential novel drug target. However, there are no known natural agonists, and small compounds that modulate MscL activity are just now being identified. Here we describe a small compound, K05, that specifically modulates MscL activity and we compare results with those obtained for the recently characterized MscL agonist 011A. While the structure of K05 only vaguely resembles 011A, many of the findings, including the binding pocket, are similar. On the other hand, both *in vivo* and molecular dynamic simulations indicate that the two compounds modulate MscL activity in significantly different ways.

## Introduction

Multi-drug resistance (MDR) in pathogenic bacteria is a major threat to human health. In 2013 the Centers for Disease Control (CDC) of US declared that the human race is now in the “post-antibiotic era,” and in 2014, the World Health Organization (WHO) warned “the antibiotic resistance crisis is becoming dire” [1, 2]. Multidrug resistant bacteria have been declared a substantial threat to U.S. public health and national security by the Infectious Diseases Society of America and the Institute of Medicine, as well as the federal Interagency Task Force on Antimicrobial Resistance. Nearly two million Americans per year develop healthcare-associated infections, resulting in 99,000 deaths, most due to antibacterial resistant pathogens [1, 3]. However, there are relatively few novel antibiotics being developed [4]. Thus, fighting MDR bacterial infections in patients is becoming increasingly difficult [3, 5]. New drugs and, more importantly, novel targets are essential.

In bacteria, mechanosensitive (MS) channels appear to play the role of emergency release valves. Briefly, when bacteria are exposed to high osmolarity they transport (K^+^, glutamate, betaine, and proline) and synthesize (glutamate, trehalose, proline and betaine) solutes to balance the increase in external osmolarity to keep cell turgor high, a requisite for cell growth and division. When the osmotic environment acutely decreases, water rushes in, turgor increases, and cell integrity is threatened. MS channels release cytoplasmic pressure by allowing the rapid efflux of cytosolic molecules from the cell [6]. There are two families of MS channels found in bacteria: the mechanosensitive channel of large conductance, MscL, and small conductance, MscS. Both appear to directly sense tension in the membrane [7, 8]. MscS channels and its homologues are more sensitive, open a smaller pore, may be more regulated [9, 10] and appear to lead to a more controlled efflux; a single bacterial species may have more than one homologue expressed. On the other hand, the majority of bacteria, including most pathogens, contain a single copy of *mscL*, which is highly conserved between species (see [11] for more discussion and sequence alignment). MscL is thought to be the last-ditch effort for a bacteria to survive osmotic downshock [6, 12]

The MscL channel has the largest gated pore known, is thought to be able to pass not-so-small proteins including thioredoxin, elongation factor Tu and DNAK ([13, 14] and see [15]), and is estimated to be on the order of 30Å in diameter [16]. Thus, it is not surprising that forward genetic experiments revealed that MscL channel mutants that are more sensitive to membrane tension and appear to gate inappropriately *in vivo* lead to bacterial slowed growth, or even decreased viability [17, 18].

Several MscL properties such as its conservation, being upregulated during the stationary phase and the fact that the inappropriate opening of its 30Å pore can decrease bacterial growth and viability, make this channel an ideal antibiotic target. This led us to the identification MscL agonists for their potential as antibiotics with the novel mechanism of essentially permeabilizing the bacterial membrane by the opening of its big pore. We thus performed a high throughput screen (HTS) to identify compounds that slowed bacterial growth in a MscL-dependent manner [19]. The goal was to find compounds that gated the MscL channel inappropriately and could be used to study channel gating and potentially serve as antimicrobial agents. Surprisingly, four known antibiotics, with other known mechanisms of action, were identified in the screen. Analyses demonstrated that some antibiotics appear to gate MscL and use it as a pathway to cross the cytoplasmic membrane [19]. The best characterized is dihydrostreptomycin (DHS) [20], where it appears that not only is MscL a primary way the bulky and multi-charged DHS molecule passes across the membrane, but it is also responsible for a DHS-dependent K^+^ flux from the cell that was observed prior to any decrease in viability [21]. It was these findings that hinted that MscL agonists may specifically permeabilize bacterial membranes to allow access of common antibiotics and could potentially be used as an adjuvant bypassing some forms of antibiotic resistance. We also identified several novel compounds not known to have antimicrobial activities, and have begun to characterize them [11, 22]. Here, we characterize a new novel small-molecule compound that we coin as K05 that directly binds to and modulates MscL gating. We find that it has the characteristics anticipated for a MscL-specific agonist, including slowing bacterial growth of several bacterial species, increasing the potency of common antibiotics, and decreasing the viability of stationary cultures. We compare and contrast this compound with what is known for others that gate the MscL channel.

## Results

### Small compound, K05, decrease *E*. *coli* growth and viability of stationary cells in a MscL-dependent manner by directly activating Eco-MscL channels

**Fig 1:**
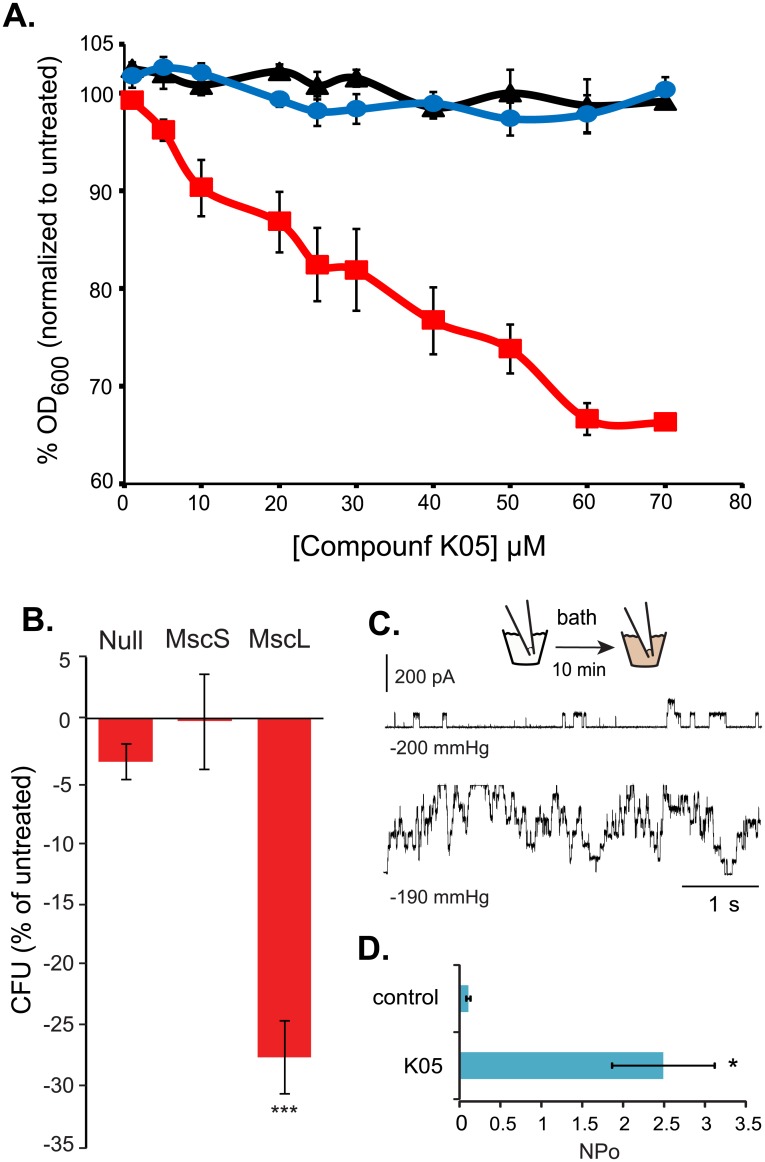
K05 affects both growing and stationary *E*. *coli* cultures in a MscL-dependent manner. (A) percentage of growth (OD_600_), normalized to untreated samples, for K05 treated (red) relative to non-treated (blue) in Eco-MscL-expressing cultures; the *ΔmscL* is shown in black. (n = 3–4) (B) The percent reduction in CFU’s after treatment with 80 μM of compound K05 after cultures have reached stationary phase. (n = 4) *** p<0.0004 by unpaired Student t-test. (C) Representative traces of Eco-MscL channels recorded by patch clamp experiments of native membranes. MscL channel activity is shown before (upper trace) and after (lower trace) addition of 50μM compound K05 to the bath. (D) Quantification of the effect of K05 on the probability of opening of MscL (NP_o_). (n = 6) p<0.02 paired Student t-test.

The K05 compound was identified by an HTS designed to look for agonists to the MscL channel [19]. To confirm that this compound decreased cell growth in a MscL-dependent manner, we assayed the influence of K05 on cell growth in a Δ*mscL E*. *coli* strain, and one expressing the MscL channel. As shown in Fig 1A, K05 decreases *E*. *coli* cell growth in a dose-dependent manner only when MscL is expressed.

MscL is constitutively expressed, and one study even suggests it is up-regulated in stationary cultures [23]. As a channel, it also does not require any metabolic energy to gate, only a stimulus. Thus, in theory the gating of MscL in stationary cells could release metabolites required to achieve a renewed growth phase, decreasing the viability of cells within the culture. We therefore tested quiescent/stationary cultures for their sensitivity to the K05 compound. As shown in Fig 1B, when stationary cultures were treated with the K05 compound, a significant decrease in viability was noted. This appears to be MscL specific because MscL-null cells, and those expressing only the other major *E*. *coli* MS channel, MscS, did not show this decreased viability. Because both MscL and MscS normally sense membrane tension, these data support the notion that K05 directly interacts with the MscL channel and is not simply adding tension to the membrane.

We used electrophysiology to test if K05 increased the activity of the MscL channel. We tested the MscL activity directly in *E*. *coli* inside-out patches of native membranes as previously described [24]. As seen in Fig 1C and 1D, compound K05, when added to the bath, significantly increased the activity of channels given a threshold membrane-tension stimulus.

### K05 treatment leads to cellular efflux of potassium (K^+^) and glutamate of *E*. *coli* MscL-expressing cells

It is known from several studies that the inappropriate gating of MscL leads to fluxing of K^+^ and glutamate from the cytoplasm [17, 20]. We therefore tested for K^+^ and glutamate remaining intracellular after K05 treatment. We found that K05 treatment led to a 38% reduction of K+ (p<0.0001) and 16% reduction of glutamate (p<0.01) only for MscL-expressing cells; no significant reduction in either osmoprotectant was observed in a MscL-null strain. These data are plotted in S1 Fig.

### Compound K05 increases the potency of common antibiotics when used at sub-threshold concentrations

**Fig 2:**
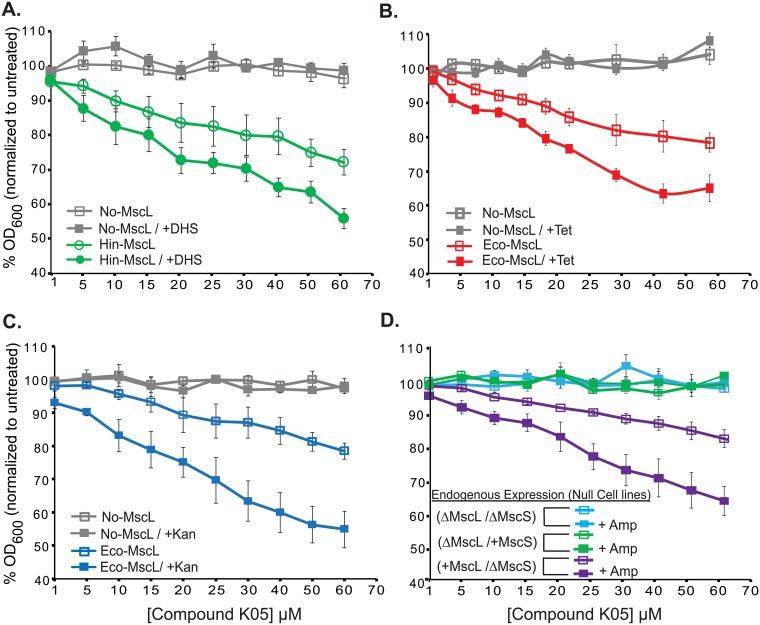
K05 increases the potency of common antibiotics. Concentration dependent effect of K05 in: (A) *E*.*coli* MJF455 cultures carrying empty plasmid or expressing (Hin-MscL) grown in the presence or the absence of 2.25 μM DHS. (B) *E*.*coli* MJF455 cultures carrying empty plasmid or expressing Eco-MscL grown in the presence or the absence of 1 μM kanamycin or (C) 0.5 μM tetracycline. (D) Minimal inhibitory concentration curves for 2 μM ampicillin for *E*. *coli* strains MJF367, MJF455 and MJF451 are shown as indicated. Note that MscL and MscS channels are expressed at endogenous protein levels. Values represent the mean of three experiments and error bars are the SEM. (n = 3).

MscL opens a very large channel upon activation, and in theory could allow cytoplasmic access for common antibiotics by essentially permeabilizing the bacterial membrane. As a first assay to determine if K05 could accelerate the passage of known antibiotics into the cytoplasm, we tested if it could enhance dihydrostreptomycin (DHS) potency. A complication is that the *E*. *coli* MscL channel (Eco-MscL) is one of the primary pathways for DHS to enter the cell [20]. However, the *Hemophilus influenza* MscL (Hin-MscL) does not bind well to DHS [20]. We therefore heterologously expressed the Hin-MscL in an *E*. *coli* MscL-null strain. As seen in Fig 2A, combinatorial treatment of 2.25 μM DHS, a normally sub-threshold concentration, and varying concentrations of compound K05 with *E*. *coli* cells expressing Hin-MscL led to much larger decreases in growth than that observed with treatment of compound K05 alone.

We next tested to see if K05 could increase the potency of a sub-threshold concentration of the antibiotics tetracycline (Tet) and kanamycin (Kan). When K05 was used in combination with either of these antibiotics, a much larger reduction in growth was observed in an Eco-MscL-dependent manner (Fig 2B and 2C). Note that while both DHS and Kan are in the aminoglycoside family of antibiotics, Tet is of another family, demonstrating the general nature of the phenomenon. Together, these data suggest that K05 holds promise to be used as an adjuvant, permeabilizing the membrane and facilitating antibiotics access to the *E*. *coli* cytoplasm.

Ampicillin (Amp) does not require cytoplasmic access but compromises the cell wall integrity, and thus we speculated that even subthreshold concentrations of Amp could potentially increase K05 efficacy. Since our plasmid, pB10d, confers resistance to ampicillin (Amp), in order to test this antibiotic, we modified our assay by utilizing null strains, and the MscL endogenously-expressing parental strain. Indeed, K05 efficacy was increased, even for endogenous levels of MscL (Fig 2D).

**Fig 3:**
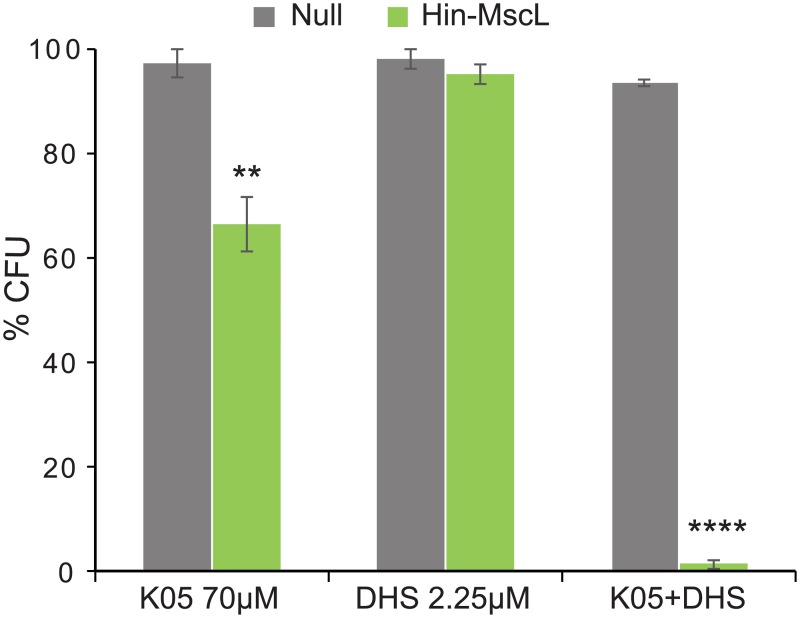
MscL dependent decrease in viability in the presence of K05 and DHS. Viability of bacterial cultures treated with 70 μM compound K05, 2.25 μM DHS or a combination of the two. Values represent the percentage of colony forming units (CFU) of the treated, relative to the untreated samples. *E*.*coli* MJF455 cultures carrying empty plasmid (null grey) or expressing Hin-MscL (green) are shown. (n = 4) **p<0.002, ****p<1.8 10^−10^ Student t-test unpaired vs empty plasmid.

DHS is a cidal antibiotic, leading to decreased viability. Hence, the decrease in OD_600_ observed should be coupled with a significant decrease in viability. Therefore, viability was also tested of three conditions: K05 (70 μM), DHS (2.25 μM) and a combination of the two at these concentrations, for both empty plasmid and Hin-MscL. There was no reduction in viability with DHS alone, and a significant but modest reduction for K05 alone. However, the combination of the two showed a greater than 98% reduction in viability for the Hin-MscL expressing bacteria (Fig 3).

### Compound K05 decreases growth of cultures and viability of stationary phase cultures of pathogenic bacterial models in a MscL-dependent manner

**Fig 4:**
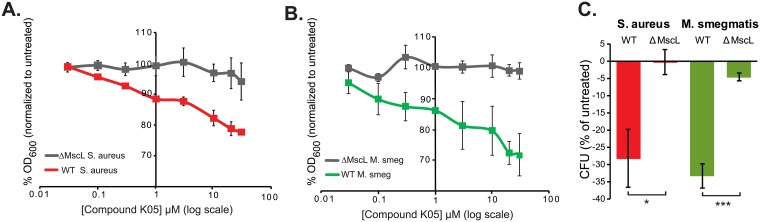
K05 affects both growing and stationary cultures of pathogenic bacterial models in a MscL-dependent manner. Growth inhibition by increasing concentrations of compound K05 are shown for (A) *S*. *aureus* R4220 WT (red) and Δ*mscL* R4220 S. aureus (grey) strains and (B). *M*. *smegmatis* MC2155 WT (green) and MC2155 Δ*mscL* (grey). Curves represent the percentage growth (OD_600_) of K05 treated, relative to untreated. Values represent the mean SEM of at least three experiments n = 3 (C). Viability of stationary phase cultures after treatment with 80 μM of compound K05. Values represent the percent reduction in CFU’s normalized by the untreated shown for the *S*. *aureus* R4220 WT and null (red) and *M*. *smegmatis* MC2155 WT and null (green) Note that WT stains are expressing endogenous levels of MscL. *p<0.03 (n = 4); ***p<1.5 10^−5^ (n = 7) Student t-test unpaired.

MscL-null strains of two species of bacteria that are used as pathogenic bacterial models have been generated: *Staphylococcal aureus* (*S*. *aureus*) strain R4220 [25] and a species often used as a model system for *Mycobacterium tuberculosis* (*M*. *tuberculosis*), the non-pathogenic *Mycobacterium smegmatis* (*M*. *smegmatis*) [11]. We tested these strains for MscL-dependent sensitivity to the K05 compound. As seen in Fig 4A and 4B, both strains showed a MscL-dependent decreased growth upon K05 treatment.

We also tested the ability of K05 to decrease the viability of these species when stationary phase cultures were treated. As seen in Fig 4C, both strains also showed a MscL-dependent significant decrease in viability of stationary non-metabolizing cultures.

### Compound K05 increases the potency of tetracycline in *S*. *aureus* and *M*. *smegmatis* cultures

**Fig 5:**
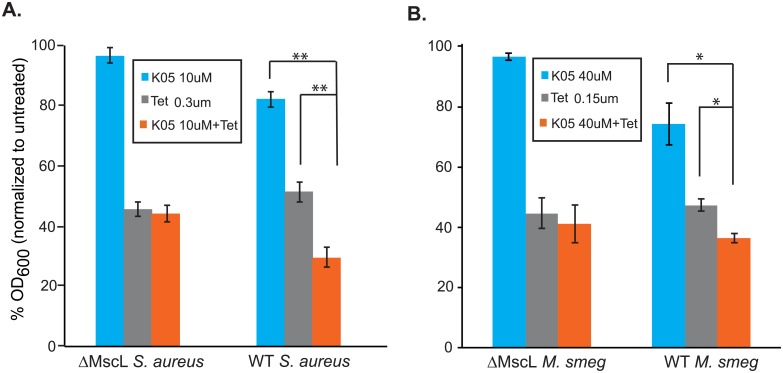
K05 increases the potency of tetracycline in a MscL-dependent manner. Two model systems for pathogenic bacteria, *S*. *aureus* (A) and *M*. *smegmatis* (B) were treated with the K05 compound, Tet or a combination of both. Values represent bacterial growth (OD600) as a percentage of the untreated. **p<0.005 (n = 3–4); *p<0.02 (n = 3) Student t-test paired.

We next tested the two pathogenic model species to see if K05 would increase the potency of a threshold concentration of Tet. As seen in Fig 5, both of these species showed MscL-dependent modest but significant decreases in growth when Tet and K05 were combined.

### K05 binds to a well-studied cytoplasmic binding site at the subunit interface

**Fig 6:**
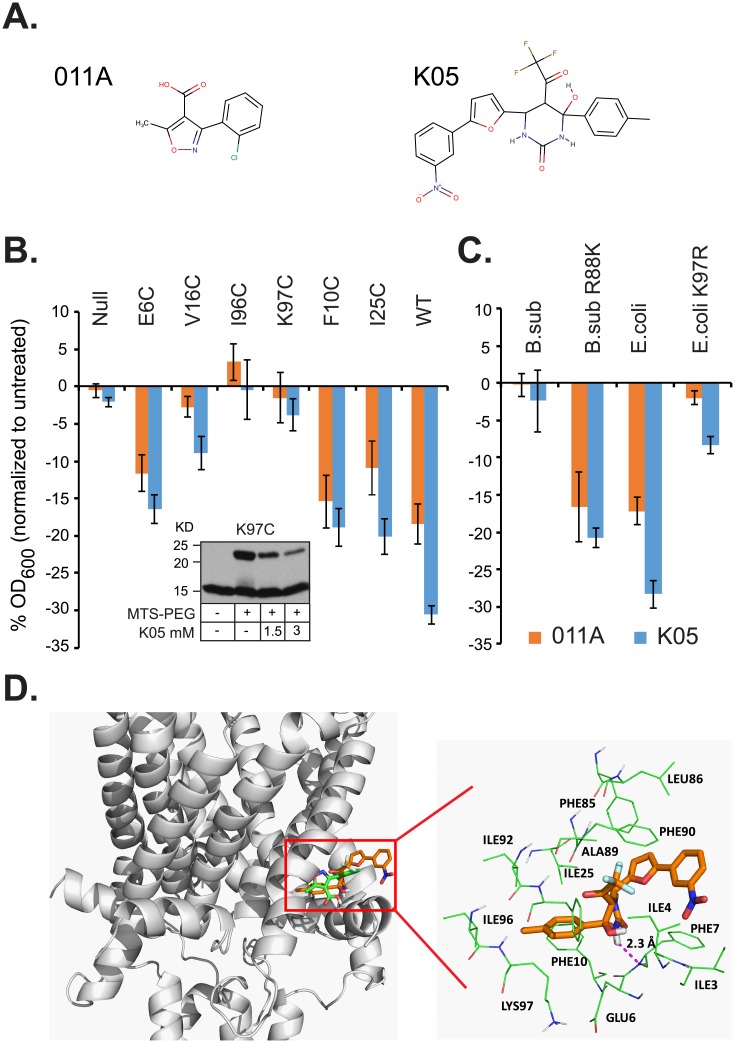
Compounds 011A and K05 show a similar binding profile in the Eco-MscL channel. (A) Chemical structures of compounds 011A and K05 are shown as stick figures. (B) Reduction in bacterial growth (OD_600_) for cultures of the MJF455 strain carrying empty plasmid (null), or expressing Eco-MscL, and cysteine mutants **Inset**: Compound K05 blocks the binding of MTS-PEG5000 to Eco-MscL K97C mutant in a dose-dependent manner. (C) B. subtilis-MscL constructs treated with compounds 011A (orange) and K05 (blue) at 60uM relative to non-treated Values for B and C represent the mean of four experiments and error bars are the SEM. (D) Binding pocket of the K05 (brown) and 011A (green) compounds as determined by the Sybyl program (left) and the key residues interacting with compound K05 (right).

The observation that K05 influences channel activity in patch clamp when applied to the cytoplasmic side of the protein implies that either the binding site is in the center of the pore, which is accessible to either side, or, perhaps more likely, K05 can easily cross the membrane. This finding, and many of the findings of the compound decreasing growth, and decreasing viability of stationary cultures, are reminiscent of another compound, 011A, that we recently reported [11, 22]. On the other hand, while K05 and 011A have some vague structural similarities, they are quite distinct (Fig 6A). This difference between the two compounds is rooted in the basic molecular properties. Unlike 011A, which is an acid, K05 is a neutral molecule and it has no violation of the Lipinski’s “Rule of 5”: molecular weight = 489, ClogP = 4.47, numbers of H-bond acceptors and donors are 9 and 3, respectively. K05 also has a high membrane permeability with a calculated *logP*_*eff*_ of 3.99 using the GastroPlus software (https://www.simulations-plus.com).

The binding site for 011A was previously determined four independent ways: searching for partial suppressors in a cysteine library we previously generated (Eco-MscL contains no endogenous cysteines) [26–31], performing a competition assay between a the compound and a thiol reaction using methoxypoly(ethyleneglycol)-5000-amidopropionylmethanethio-sulfonate (MTS-PEG5000), assaying and modifying the proposed binding site of orthologues and finally, molecular docking. We used this array of assays again here to determine if compound K05 had a binding site that overlapped with 011A.

We assayed cysteine mutants in and around the region of 011A binding site with K05 to see if the same mutants were partial/full suppressors. As seen in Fig 6B, Eco-MscL cysteine mutants show the same suppressor profile for 011A and K05: I96C and K97C are almost complete suppressors, while V16C is only a partial suppressor of the slowed growth of *E*. *coli* cultures. In contrast, only small changes are seen with E6C, F10C and I25C, the latter of which is the primary suppressor and part of the binding site for DHS [20].

Given the similarity in the suppressor profile of cysteine mutants, we wanted to further validate the hypothesis that 011A and K05 truly had similar binding sites. We have found that using purified cysteine mutants and MTS-PEG5000, that a competition assay with small compounds is reliable for determining a binding site; this has been used for both DHS [20] and 011A [22]. Using this assay for a detergent-solubilized and purified Eco-MscL K97C mutant, we found that compound K05 can compete for and inhibit the modification of the cysteine by MTS-PEG5000, as indicated in the Fig 6B **inset**. A concentration-dependent decrease of the upper PEG5000-modified band was observed after the addition of K05. This was also previously observed for the 011A compound [22].

The MscL channel from *Bacillus subtilis* (*B*. *subtilis*) is one of the very few orthologues that does not have a K at the equivalent to the *E*. *coli* 97 residue position, but instead has an R. When the *B*. *subtilis* MscL is heterologously expressed in an *E*. *coli* MscL-null strain, the strain is also largely resistant to the K05 compound (Fig 6C). These data contrast those obtained for MscL orthologues from other organisms that have a K at the equivalent of position 97, including *Clostridium perfringens*, *Hemophilus influenza*, and *S*. *aureus* (S2 Fig). As seen in Fig 6C, mutating the *B*. *subtilis* residue that is equivalent to the *E*. *coli* 97 site, R88, to K (B.sub R88K) makes this orthologue now sensitive to compound K05 treatment. In contrast, mutating the Eco-MscL to K97R significantly decreases the sensitivity of the Eco-MscL to compound K05 treatment. Again, as shown (Fig 6C and S2 Fig), these data mirror those previously observed for compound 011A [22].

To better compare and contrast the binding of compounds 011A and K05, we first performed flexible-ligand docking following the protocol previously used [22]. The docking score, -7.22 is comparable to that of 011A, -7.09 kcal/mol. As shown in Fig 6D, the nitrobenzene fragment sticks out the binding pocket and penetrates deep into the lipid, a major difference from 011A. A hydrogen bond is formed between the compound K05 ligand and the main chain of F7. However, both the 011A and K05 have contacts with F10 and I25. The second-best docking pose, which has a docking score of -6.66 kcal/mol, is essentially similar to the best docking pose except that the nitro functional group pointing to an opposite direction (S3A Fig). Two more docking poses, Docking Poses 3 and 4, which have docking scores of -6.55 and -6.48 kcal/mol, are distinct from the best two docking poses. Particularly for Docking Pose 3, the K05 structure is flipped and the nitro function group is residing inside of the binding pocket (S3B Fig). The detailed interactions between Eco-MscL and K05 are shown in Fig 6D and S4, S5, S6 and S7 Figs.

Together, the data strongly suggest that although compounds 011A and K05 do not have a noticeable strong similarity in their scaffold, they appear to bind to a common binding site at the interface of two subunits, an area that has been shown previously to move in a very defined way upon gating [29].

### Molecular dynamics (MD) simulations strongly support the hypothesis that K05 directly binds to and modulates MscL activity

To better characterize the protein-ligand interaction, we performed conventional molecular dynamics (MD) simulations to investigate the dynamics of the ligand binding for three distinct docking poses, namely, Docking Poses 1, 3 and 4. For Docking Pose 1, the system reached equilibrium phase after 20 nanoseconds (ns) according to the Root-mean-square deviation (RMSD) ~ Simulation Time curve for the secondary structures (red curve of S8 Fig). We identified two conformation clusters based on the RMSD curves for the ligand. The green curve, obtained by performing least-square (LS) fittings, represents the conformational change of the ligand during the MD simulation. In contrast, the blue curve (No Fit) measures not only conformational change, but also the translational and rotational movement of the ligand. The representative conformations of the two clusters are shown in S3D and S9 Figs. For both conformational clusters, the nitrobenzene group penetrates into the lipid. The major difference of the ligand conformations lies in that there is ~180 rotation along the single bond linking the nitrobenzene and furan functional group, otherwise, the two representative conformations are very similar. It is pointed out that the RMSDs of the ligand shown in S8 Fig are much higher when the aforementioned rotation occurs than otherwise. A ‘smart’ program, which can automatic detect the equivalent atoms, is desired to overcome the defect of the current LS fitting program for RMSD calculations. For Docking Poses 3 and 4, the MD trajectories are less stable than that of Docking Pose 1 as indicated by the RMSD ~ Simulation Time curves (S10 and S11 Figs). For Docking Pose 1, the curve for the secondary structures are overall below 2 Å, while the curves are mostly above the 2 Å for Docking Poses 3 and 4. As long as the RMSD curve of the whole Eco-MscL is considered, the difference is more obvious.

After the system reached equilibrium, 135 and 1350 MD snapshots were evenly recorded for the MM-PBSA (Molecular Mechanics-Poisson Boltzmann Surface Area) [32–36] free energy calculation and MM-GBSA (Molecular Mechanics-Generalized Born Surface Area) [33–35] free energy decomposition, respectively. There are many approaches for free energy calculations including the pathway-based approaches, such as free energy perturbation and thermodynamic integration, [37, 38], the aforementioned endpoints-based methods, MM-PB/GBSA, and potential of mean force (PMF) based methods [39, 40]. Although MM-PBSA itself is not theoretically rigorous as the FEP/TI, [41] it is widely used these days due to its relatively less computer-resource demanding and easier to run. One can achieve satisfactory prediction in relative free energy calculations for many systems with the MM-PBSA method [32, 42]. In this work, we applied the MM-PBSA method to identify the most favorable binding mode from a set of docking poses following an established protocol [43].

We first calculated the MM-PBSA free energies for the Eco-MscL/K05 complex to identify which binding mode was most favorable. As shown in S1 Table, the binding mode represented by Docking Pose 1 has the smallest MM-PBSA free energies for all the three considered lipid dielectric constants. This result is consistent with the RMSD ~ Simulation plots which reveal Docking Pose 1 is the most stable. If not specifically pointed out, the discussion is referred to Docking Pose 1 in the remaining text. Sequentially, we conducted MM-PBSA binding free energy calculations for the best docking pose. As shown in S2 Table, the MM-PBSA binding free energy is slightly lower for the first conformation cluster when ε_lip_ = 1 and 2, while it is lower for the second conformation cluster when ε_lip_ = 4. The overall binding affinity is worse than that of 011A, which is -10.32 kcal/mol using a lipid dielectric constant of 4. Note that although the binding free energy for Pose 3 is much smaller than that of Pose 1, it is not a favorable binding pose as its MM-PBSA free energy of the complex is much worse than that of Pose 1 (S1 Table). As an endpoint method, MM-PBSA results are affected by the setting of parameters [32]. The desolvation penalty decreases with the increase of lipid dielectric constant, leading to increase of binding affinities. Although, we did not measure the inhibitor constant, the calculated binding affinities, -6.55 kal/mol with ε_lip_ = 1, -7.90 kcal/mol with ε_lip_ = 2 and -8.37 kcal/mol with ε_lip_ = 4, are in reasonable range. We performed MM-GBSA free energy decomposition to identify hotspots of the protein-ligand binding. The hotspots, which have interactions with the ligand better than -1.0 kcal/mol, include residues F7, F10, F25, F85, L86, A89 and F93. V16 has an interaction of -0.90 kcal/mol (S3 Table). The free energy decomposition results only partially explain the cysteine mutation profile of K05. It is noted that the simulations and analyses for 011A were detailed in two recent publications [11, 22].

In one of the two publications, we have demonstrated that compound 011A could facilitate the passage of dihydrostreptomycin (DHS) in virtual “passing through” experiments in which the compound was bound to MscL and the facilitation of the passage of DHS through the channel was monitored [11]; note that the passage was accelerated by external electric field (EFF) which was applied only to DHS. We therefore further studied the gating mechanism of K05 by performing such passing-through experiments through nonequilibrium MD simulation. The passing-through events were observed by measuring the distance between the geometric centers of DHS and five lysine residues (106, 242, 378, 514 and 650) of the pentameric complex. Interestingly, we found that there is a substantial difference between the gating mechanism of K05 to that of 011A. 011A has a stronger effect on inducing channel opening and the EEF needed to induce the passage of DHS is much smaller. We have observed 18 successful passing-through events among 36 independent MD runs with an EEF of 0.1 Volt/Å [11]. However, no successful passage of DHS was observed for K05 with the same EEF among 14 independent MD runs. Only after the EEF was increased to 0.15 and 0.2 Volt/Å we could observe the successful passage of DHS. As shown in S12 Fig, DHS passed through the Eco-MscL 15 times in a successful virtual passing-through experiment using an EEF of 0.2 Volt/Å. Next, we calculated the residue-based channel radius parameter, *r*, which is defined as the mean distance between the geometric center five equivalent residues (residues at the same position of the five chains) and the geometric centers of individual residues for the collected MD snapshots. The larger the *r*, the more open the channel is at the positions of the five residues. The channel radius parameters as well as their standard errors were listed in S4 Table for seven scenarios, which are MscL only (I), MscL/011A (II), MscL/K05 (III), MscL/011A/DHS with channel open induced by EEF (IV), MscL/K05/DHS with channel open induced by EEF (V), MscL/011A with channel open without DHS (VI), and MscL/K05 with channel open without DHS (VII). For Scenarios I to III, and VI to VII, MD snapshots come from conventional MD simulations. In contrast, the snapshots of Scenarios IV and V were collected from the “passing-through” experiment. The change of *r* parameters to the references value of Scenario I, Δr, are shown in S13 to S15 Figs for Residues 10–40. The relative positions of residue 10–40 are shown in S16 Fig. As shown in S13 Fig, 011A has a better capacity to open MscL channel than K05, which is consistent to the observation that a smaller EEF is required to induce DHS passage. As to Scenarios 4–5, when the MscL channel was opening, allowing DHS passage, K05 opened the channel wider than 011A at the positions of residues 21–32. Note that in none of these cases does the channel achieve the fully open state predicted with a 30Å pore because of the time limitations of the simulations. However, the results do suggest that the primary and initial conformational changes induced by compound 011A binding is quite different than that for compound K05 binding; the former seems to affect the cytoplasmic “gate” more prevalently, while the latter affects the pore “vestibule” more in the center and periplasmic region of the first transmembrane domain (TM1).

We then investigated how well K05 can maintain the channel-opening conformation. We performed 100-nanosecond conventional MD simulations from a channel-opening conformation after removing DHS. As shown in S17 Fig, both 011A and K05 can maintain MscL channel-open conformations for 80 ns if a threshold of 3.0 Å for RMSDs of the secondary structures is applied. However, the mainchain RMSDs of helices are relatively smaller for compound 011A than for compound K05, suggesting 011A can better maintain the channel-opening conformation than K05. This result is consistent with the observation that the *Δ r* profiles of Scenarios 4 and 6 are similar. In contrast, the MscL channel narrowed, closing significantly for compound K05 as suggested by *Δr* profiles of Scenarios 5 and 7. As shown in S15 Fig, the channel constriction occurred around residues 10–22 (the cytoplasmic side). It is noted that although we only performed 100 ns MD simulations, the system needs much longer time to restore to the channel-close conformation from the channel-open conformation induced by the passage of DHS. In sum, these data show that compounds 011A and K05 have significantly different mechanisms of regulating MscL channel gating with compound 011A opening the channel more fully at the gate and achieving a more stable opening structure.

We then performed MM-GBSA binding free decomposition for the Scenario 7. We found that the binding profile is quite different from that of Scenario 3. The identified hotspots were listed in S3 Table. The binding profile of Scenario 7 may help to explain some of the findings of the suppressor profile of the cysteine mutants (Fig 6B). The contribution of F10 and I25 to the ligand binding is negligible. Although the interaction energy between V16 is small, its four neighbors, G14, N15, V17 and D18 are all hotspot residues. Therefore, mutation of V16 could interfere with the binding of K05 to Eco-MscL at the channel-opening state.

## Discussion

Given that MscL is the largest known gated pore, it is not surprising that when MscL gates at inappropriate times, it is detrimental to the cell [17, 18, 26, 27, 30, 31]. This finding alone suggested that MscL could be a drug target for isolating novel antibiotics. However, there are no known endogenous agonists to the MscL channel, and early studies were only successful at discovering non-specific modulators.

Historically, it was found that amphipaths that intercalate asymmetrically into the membrane could modulate or gate bacterial osmosensors and MS channels [44–46], and indeed they have also been shown to modulate MscL activity [47]. However, these compounds simply change the pressures within the membrane and are therefore non-specific [48]. In one study [49], a compound found through in silico screening was found to inhibit growth of MscL-expressing cells, but it also has some influence on cells expressing MscS, suggesting that at least some of its action was non-specific, presumably through the membrane. It also still inhibited the growth of cells not expressing MscL or MscS, albeit at higher concentrations, demonstrating that it has at least one other mode of action [50]. Thus, until recently, there were no agonists that directly bound to MscL and modified its activity.

We tried to bypass some of these issues by using a HTS approach. In our search for MscL-specific agonists we performed a secondary screen and removed candidates that effected slowed growth on MscS-expressing cells [19]. To our surprise, we identified some known antibiotics. Subsequent studies confirmed that the influences on growth and viability of the aminoglycosides spectinomycin and DHS were specific to MscL; MscS expression had no influence. But MscL expression only increased the potency of these antibiotics, consistent with the primary mode of action being the inhibition or disruption of protein synthesis. In a subsequent study, we demonstrated that DHS does indeed directly binds to, modulates, and passes through the MscL channel pore, appearing to be one of the major pathways for cytoplasm access [20].

More recently, novel compounds identified in the HTS have begun to be characterized. One of them, compound 120, is a sulfonamide compound that genetic experiments have confirmed has the common primary mode of action of antimicrobial sulfonamides of inhibiting folate synthesis; presumably it was identified by the HTS because, like DHS, it uses MscL as a primary pathway for cytoplasmic access [22]. However, another sulfonamide compound identified, 011, did not appear to have any action through the folate pathway, and in fact, had increased potency when the sulfonamide portion of the structure was removed, yielding a compound coined 011A [22]. We thus continued to study the 011A compound, its ability to affect the growth of other bacterial species and increase potency of commonly used antibiotics [11].

Several independent lines of evidence clearly indicate that the K05 binding site overlaps with that previously found for the 011A compound [22]. These include molecular docking, searching for partial suppressors in a cysteine library we previously generated, performing a competition assay between the compound and a thiol reaction using methoxypoly(ethyleneglycol)-5000-amidopropionylmethanethio-sulfonate (MTS-PEG5000), and assaying and modifying the proposed binding site of orthologues. While 011A appears to have tighter binding, as determined by MM/PBSA computational analysis, K05 opens the channel wider, but not in a stable manner. This shared binding pocket, which is at the interface of two subunits, has been shown to be functionally important in the gating process [29, 30, 51, 52].

We used the same *in vivo* assays to study the K05 compound that we used to characterize the 011A compound. Although the compounds have only vague structural similarities, they led to what are essentially the same results. There are also no obvious differences seen in the electrophysiological recordings. On the other hand, it is curious that the K05 compound shows better efficacy in the assay for the inhibition of cellular growth (35% decreases with K05, but only 25% decreases with 011A), but less efficacy in decreasing the viability of stationary cells (about 30% for K05 but 50% for 011A). We have established a set of computational and experimental means to study and develop more efficient MscL channel modulators which also indicate significant differences. In our MD simulations we find that although 011A binds tighter, has a greater probability of allowing DHS to pass, and maintains the opening structure longer, K05 causes a more dramatic yet transient change in channel opening, especially toward the channel vestibule. These results suggest that although the 011A and K05 compounds share a binding pocket, they modulate the channel in different ways–perhaps K05 leads to a more sensitive channel, but with more rapid kinetics due to the lower binding affinity, while the tighter binding of 011A leads to a more fully open channel, thus allowing larger (and, in stationary cultures, less replaceable) chemicals/components into or out of the cytoplasm.

In future studies, it will be of interest to modify the structure of K05 to further improve its binding affinity and select for compounds that fully open the channel. During MD simulations of the first docking pose, we observed that the nitro functional group penetrates deep into lipids. This is perhaps not surprising as nitro functional groups can reside in both hydrophobic and hydrophilic environments as shown by studies on the hydrogen bonding and membrane permeability of nitro functional groups conducted a half-century ago [53–55]. Given that nitro groups can have toxicity issues [56], it may be worthwhile to test other groups such as isopropyl. Prior to costly chemical synthesis, the binding and potency of newly designed compounds, as well as their ability of opening MscL channel, can be initially evaluated using our established computational protocols.

## Conclusions

We have now studied two compounds that appear to have MscL gating as a primary mode of action: the previously characterized compound 011A [11, 22], and K05, which is characterized here. Both compounds inhibit growth, inhibit viability of stationary cultures, and appear to allow passage of antibiotics and osmoprotectants into and out of the cell, respectively. However, physiologically, and in MD simulations, there are some differences in the effects of these compounds, suggesting they modulate channel activity in subtly different ways: one likely interpretation is that compound K05 leads to a more dramatic change in channel opening, but the partial or full openings cannot be maintained, which probably prohibits larger molecules to pass; 011A binding tighter and leading to more full openings that are maintained longer, allowing these larger molecules to pass. The reason this is not seen in patch clamp may be due to differences between patch and *in vivo*, such as the membrane potential (-20mV in patch, approx. -160mV *in vivo*), or perhaps that the asymmetry between headgroups seen in the bilayer *in vivo* is lost in patch. Interestingly, the binding pocket for both 011A and K05 lies between two protein domains: an N-terminal surface helix, S1, and the cytoplasmic portion of the second transmembrane domain from a different subunit. These domains have been shown to slide against one another as the channel opens; inhibiting this movement locks the channel closed [29]. Our data now suggest that disrupting this interaction by binding a small compound in this region can facilitate this movement, thus making the channel more likely to open. This protein-protein interface between subunits is thus a target binding pocket for finding additional compounds in the future that are MscL modulators, possibly even agonists with higher affinity and/or greater efficacy.

## Materials and methods

### Strains and cell growth

The following bacterial strains were used in this study: *S*. *aureus* R4220 and R4220 ΔmscL [25], *M*. *smegmatis* MC2155 and MC2155 ΔmscL [11], *E*. *coli* MJF367 (ΔmscL::Cam), MJF451 (ΔmscS) [57], *E*. *coli* MJF455 (ΔmscL::Cam, ΔmscS) [57] and *E*. *coli* strain MJF612 (Frag1 ΔmscL::cm, ΔmscS, ΔmscK::kan, ΔybdG::aprΔ) [58]. Note that mostly endogenous expression levels (not overexpression) was evaluated in this study unless specifically noted. For expression in the *E*. *coli* strains MscL constructs using the pb10d plasmid were used. For *M*. *smegmatis* MscL was expressed in the MscL null strain using plasmid pNH02 [11].

*E*. *coli* strains were either grown in citrate-phosphate-defined media (CphM) pH 7.0, consisting of per liter: 8.57 g of Na_2_HPO_4_, 0.87 g of K_2_HPO_4_, 1.34 g of citric acid, 1.0 g NH_4_SO4, 0.001 g of thiamine, 0.1 g of MgSO_4_7H2O, 0.002 g of (NH_4_)_2_SO_4_.FeSO_4_.H2O, or K10 media consisting of per liter: 6.53g of Na_2_HPO_4_, 3.17g of NaH_2_PO_4_, 1.06g (NH_4_)_2_SO_4_, 0.75g KCL, in a shaker incubator at 37 °C, rotated at 250 cycles per minute. Ampicillin was added for strains carrying plasmid constructs (100 μg/ml) and expression induced by addition of 1 mM isopropyl-β-D-thiogalactopyranoside (IPTG) (Anatrace, Maumee, OH). *S*. *aureus* strains were grown in Lennox Broth medium (LB) (Fisher Scientific, Pittsburgh, PA) at 37 °C, and rotated at 250 cycles per minute. *M*. *smegmatis* strains were grown on 7H10 plates (BD Bioscience, Sparks, MD), made per manufacture instructions, and then in into 7H9 media consisting per liter: of 4.7 g 7H9 Difco Middlebrook powder (BD Bioscience, Sparks, MD), 4ml 50% glycerol and 0.05% tween 80, at 37 °C, and rotated at 130 cycles per min (see details in [11]).

### *In vivo* assays

Minimal inhibitory concentration curves were performed as previously described [11, 22]. Briefly, overnight cultures *E*. *coli* MJF455 carrying the indicated constructs were diluted 1:50 in CphM and grown until an OD_600_ of 0.2 was reached, expression was then induced by the addition of IPTG for 30 minutes. Cultures were diluted 1:200 in pre-warmed CphM, 100 μg/ml ampicillin, 2 mM IPTG and 100 μl added to wells of 96 well plate (Greiner bio-one, Monroe, NC) containing 100ul of CphM with K05 at two times its final concentration solubilized in sterile dimethyl sulfoxide (DMSO) (Sigma-Aldrich, St. Louis, MO), with a final concentration of DMSO at 0.9%. For endogenous expression of MscL MJF367, MJF451, MJF455, R4220 and MC2155 strains were used without the addition of antibiotics until an OD_600_ of about 0.35 was reached. Then cultures diluted 1:200 for all strains but *M*. *smegmatis* MC2155 which was diluted 1:2, in the same growth media with or without the antibiotic being tested at two times their final concentrations. The following antibiotics were used: Dihydrostreptomycin sesquisulfate (DHS) (Sigma Aldrich St. Louis, MO), kanamycin A (Kan) (Sigma Aldrich St. Louis, MO), Tetracycline Hydrochloride (Tet) (Thermo Fisher Scientific Waltham, MA) and Ampicillin Sodium Salt (Amp) (Thermo Fisher Scientific Waltham, MA). 100 μL of these mixtures were immediately added to 100 μL of media with or without compound K05 (2X final concentration), in a 96 well plate as described above. As described in [11] for agonist 011A, K05 was also added only after the antibiotics. The above plates were sealed with a sterile breathable film to prevent evaporation (Axygen, Union City, CA), wrapped in aluminum foil and placed in a 37 °C shaker, rotated at 110 Cycles per minute for 16–17 hours and OD620 was then taken with a Multiskan Ascent 354 (Thermo Fisher Scientific Waltham, MA) plate reader.

### Viability at stationary phase

Assessment of viability after treatment of stationary phase cultures with K05, was performed as previously described [11, 22]. Briefly, for the Eco-MscL construct expressed in MJF455 strain, cultures were grown from a single colony in degassed CphM with an argon gas overlay, caped and sealed. Cultures were induced at an OD_600_ of 0.2 with the addition of 1mM IPTG, argon added, sealed and grown overnight. Overnight cultures were then divided, compound K05 or mock (DMSO only) were added at 80μM with a final DMSO concentration of 0.9%, argon added and sealed in a glass culture tube for 6 hours in a 37°C shaker. Final OD_600_ was taken and cultures were then diluted 1:20 into pre-warmed CphM, serially diluted from 10^3^ to 10^6^ and liquid drops of 5 μl for each dilution were placed on pre-warmed LB plates and placed in a 37°C incubator. The next morning colony-forming units were calculated to determine cell viability, as previously described [51, 59]. For the other bacterial species, compound K05 was added at 40uM for *S*. *aureus* or 20uM for M. smegmatis and times were increased to 48 hours for the *M*. *smegmatis* MC2155 and MC2155 ΔmscL strains.

### Electrophysiology

Giant spheroplasts were generated from the *E*. *coli* strain MJF612 and used in patch-clamp experiments as described previously [24]. Excised, inside-out patches were examined at room temperature under symmetrical conditions using a buffer comprised of 200 mM KCl, 90 mM MgCl2, 10 mM CaCl2, and 5 mM HEPES pH 6–7 (Sigma, St. Louis, MO). For every data set the same patch is recorded before and after perfusion of the 50μM of K05 compound in the bath chamber. Recordings were performed at –20 mV (positive pipette). Data were acquired at a sampling rate of 20 kHz with a 5 kHz filter using an AxoPatch 200B amplifier in conjunction with Axoscope software (Axon Instruments, Union City, CA). A piezoelectric pressure transducer (World Precision Instruments, Sarasota, FL) was used to monitor the pressure throughout the experiments. Measurements were performed using Clampfit10 from Pclamp10 (Axon Instruments, Union City, CA).

### Steady state glutamate

The *E*. *coli* MJF455 strain was used carrying either the pb10d empty vector or expressing WT Eco-MscL in the same pb10d vector. Overnight cultures were inoculated from a single colony in K10 media. The next day cultures were diluted 1:100 in the same K10 media and expression was induced with 1 mM IPTG for 1 hour after an OD_600_ of 0.2 was reached. The cultures were then split in two; mock (DMSO only) or treated with compound K05 at 70 μm with a final DMSO concentration of 0.9% for all samples. Cultures were grown overnight for 17 hours in a 37°C shaker when the final OD_600_ was recorded. For each sample 6 ml was pelleted. Pellets were brought up in the same media adjusting volume for OD and sonicated for 2 min. Glutamate measurements were performed using the EnzyChrom Glutamate Assay Kit (BioAssay Systems, Hayward, CA, US) per the manufacturer’s instructions. Viability assays were done in conjunction.

### Steady state K+

The MJF455 strain was used for the K+ steady state experiments carrying either the pb10d empty vector or expressing WT Eco-MscL in the same pb10d vector. Overnight cultures grown in K10 media from a single colony were diluted 1:25 into the same media, grown to an OD_600_ of 0.2, when expression was induced with 1 mM IPTG for 1 hour. Cultures were then split into; mock (DMSO only), or treated with K05 at 70 μm, with a final DMSO concentration of 0.9%. Cultures were then grown for 17 hours in a 37°C shaker. Final OD_600_ was then recorded and the volume of cells to be used was adjusted for OD. Typical OD_600_ values ranged from 1.0 to 1.3 and approximately 950 μl-1.2 ml of the cultures were passed through a 0.45-μm filter and washed with K0 media (K10 with the KCL replaced by NaCl) having the same osmolality, without ampicillin or supplements. Filters were placed in a plastic beaker, covered with foil and dried in an 80°C oven overnight. The next day 3 ml of double distilled water was added to each beaker and K+ was measured using a Jenway flame photometer (Cole-Palmer, East Norwalk, CT).

### MTS-Peg5000 competition experiments

MscL protein purification procedure and competition experiments were performed as previously described [20]. Briefly, 5 μg of purified MscL protein in buffer containing 50 mM KPi pH 7.5, 150 mM NaCl, 4 mM DM, was added to 0.2 ml PCR tubes. For each sample the following conditions were set up (i) mock-buffer and protein only, (ii) PEG-buffer and protein, (iii) experimental-buffer, protein, plus K05 at 1.5 mM, 3 mM in 2% DMSO. Samples were incubated for 15 min at 22°C. Methoxypoly (ethyleneglycol)-5000-amidopropionyl-methanethiosulfonate (MTS-Peg5000) at 50 μM was then added to all conditions excluding mock incubated for 10 min. Reactions were then stopped by adding 20 μl of non-reducing sample buffer with a final concentration of 5 mM Iodoacidamide, ran on a 4–20% gel and western blots preformed. Blots were probed with the primary antibody, Penta-His at 1:4,000 (Qiagen, Germantown, MD.) and secondary, goat anti-mouse at 1:70,000 (Bio-Rad, Hercules, CA.).

### Molecular docking

Molecular docking was conducted as previous [22] using a representative structure of a 150-nanosecond molecular dynamics trajectory of Eco-MscL with a DHS molecule within the gated pore, described previously [20]. The binding pocket was identified by the SiteID module of the Sybyl-X2.11 software package [60]. Then flexible-ligand docking was performed for K05 with the Glide module of the Schrodinger software package following the standard procedure [61]. The top docking poses were manually examined and we found that the Top 2 docking poses are very similar and other docking poses have much worse docking scores (S2A Fig). Therefore, only the best docking pose was selected for further study.

### Molecular dynamics simulations and binding free energy calculations

There are three different types of simulations conducted in this study. First, conventional molecular dynamics simulation was performed for the Eco-MscL/K05 complex from the docking study. The simulation box consists of one copy of MS channel protein, one copy of K05, a lipid bilayer (230 POPC molecules) with 0.1 M KCl and about 32000 TIP3P [62] water molecules. For the force field parameters, the partial atomic charges of K05 were derived using the RESP program [63] to fit the HF/6-31G* electrostatic potentials generated with the GAUSSIAN 16 software package [64]. The other force field parameters of K05 came from GAFF [65]. We applied the AMBER FF14SB [66] and LIPID14 [67] force fields to model proteins and lipids, respectively. MD simulations were performed to produce isothermal-isobaric ensembles and the Particle Mesh Ewald (PME) method [68] was applied to calculate the full electrostatic energy of a unit cell in a macroscopic lattice of repeating images. For each MD simulation, the system was first relaxed to remove any possible steric clashes by a set of 2,000-step minimizations with the main chain atoms restrained using the harmonic restraint force constants which decreased from 20 to 10, 5, and 1 kcal/mol/Å^2^, progressively. After that, the system was further relaxed by a 5,000-step minimization without any constraints or restraints. There were three phases for the subsequent MD simulations: the relaxation phase, the equilibrium phase, and the sampling phase. In the relaxation phase, the simulation system was heated up progressively from 50 K to 250 K at steps of 50 K. At each temperature, a 1-nanosecond MD simulation was run without any restraints or constraints. In the following equilibrium phase, the system was equilibrated at 298 K, 1 bar for 15 ns. Finally, a 135-nanosecond MD simulation was performed at 298 K, 1 bar to produce NTP (constant temperature and pressure) ensembles. Integration of the equations of motion was conducted at a time step of 1 femtosecond (fs) for the first phase and 2 fs for the last two phases. In total, 1350 snapshots were recorded from the last phase and used for the MM-GBSA analysis and 135 snapshots were evenly selected for MM-PBSA binding free energy calculations. To investigate when the MD system reached equilibrium, we calculated RMSDs ~ Time curves for the mainchain atoms of the whole protein, the mainchain atoms of the secondary structures, and the heavy atoms of K05. For the ligand, both the LS-Fit and No Fit RMSDs were calculated. The latter were calculated directly without LS fitting after the ligand was translated and rotated using a transformation matrix which came from the LS fitting of the MscL secondary structures.

Second, we performed nonequilibrium MD simulations to study the passage of DHS through the MscL channel. The basic molecular dynamic simulation protocol was the same for described previously [20]. An external electric field (EEF) was applied to DHS to facilitate its passage. Starting from a small EEF value, 0.1 Volt/Å, we gradually increased the EEF value so that the successful passage of DHS occurred within 100 nanoseconds. Eventually, we repeated the passing-through experiments 15 times using an EEF of 0.2 Volt/ Å. Last we performed a conventional MD simulation starting from a MscL channel-open conformation with DHS removed. The channel-open conformation was selected from a virtual passing-through experiment when DHS was about to exit from the channel. By studying the RMSD ~ Time curves and the channel radius parameters, we can evaluate how well K05 can maintain the channel-open conformation. We found that K05 can maintain RMSDs for the secondary structures below 3.0 Å (the threshold) for about 80 ns. After that, the RMSDs kept increasing and we stopped simulations at 110 ns (S17 Fig).

The following is basic protocols of binding free energy calculations and decomposition analysis. For each MD snapshot, the molecular mechanical (MM) energy (E_MM_) and the Poisson-Boltzmann Surface Area (PBSA) were calculated without further minimization. Unlike the regular MM-PBSA analysis for global proteins, two external dielectrics (ε_wat_ = 80 for water and ε_lip_ = 4.0 for the lipid bilayer) were applied for this system. The membrane center offset parameter (mctrdz), which varies from snapshot to snapshot, were calculated using the coordinate centers of Eco-MscL and the POPC bilayer. The thickness of membrane was set to 18 Å, as described previously. For K05 itself, the implicit membrane option was turned off and the external dielectric constant was set to 80. The nonpolar solvation energies were calculated using solvent accessible surface areas (SAS) using the following equation: *ΔG*_*nonpolar*_ = 0.0054 × *SAS* + 0.92. The entropic term was estimated using a method described somewhere else [69]. To investigate how different values of ε_lip_ affect the binding free energy result, we also conducted MM-PBSA analysis using ε_lip_ of 1 and 2. For MM-GBSA free energy decomposition, the GB model developed by Hawkins et al. [70] was applied to take the solvent effect into account using the internal and external dielectrics of 1.0 and 4.0, respectively. All the MD simulations and the followed free energy analysis were performed using the AMBER14 software package [71].

## Supporting information

S1 Fig K05 induces a MscL-dependent reduction in K+ and glutamate steady state. Shown are results from MJF455 cells expressing an empty vector (Null) or E. coli MscL (Eco MscL). A) The reduction of potassium was measured as the percentage of K+ remaining after overnight incubation of 70 μM K05 compared to control (non-treated with K05). Error bars reflect s.e.m. n = 9, p < .00005, unpaired TTEST. B) The reduction of glutamate was similarly measured as the percentage remaining after overnight incubation of 70 μM K05 compared to control. Error bars reflect s.e.m. n = 3, p < .01, unpaired TTEST.
(PDF)

S2 Fig MscL orthologues show a similar growth restriction profile for 011A and K05. Reduction in bacterial growth (OD_600_) for cultures of the *E*.*coli* MJF455 strain carrying empty plasmid (null), or expressing Eco-MscS (MscS), *C*. *perfringens* (C. perf), *S*. *aureus* (S. aur), or *H*. *influenza* (H. infl) constructs treated with compounds 011A (orange) and K05 (blue) at 60uM relative to non-treated are shown. Values represent the mean of four experiments and error bars are the SEM.
(PDF)

S3 Fig Comparison between top docking poses and the representative conformations of the two clusters sampled by MD simulations. The Top 1 pose (docking score = -7.22 kcal/mol) is always shown as brownish sticks and three other poses are shown in greenish sticks: Docking Pose 2 (docking score = -6.66 kcal/mol2) in Panel A, Docking Pose 3 (docking score = -6.55 kcal/mol) in Panel B and Docking Pose 4 (docking score = -6.48 kcal/mol) in Panel C. The representative conformations of Clusters 1 and 2 are shown as greenish and brownish sticks (Panel D).
(PDF)

S4 Fig Binding pocket (left) and the key residues interacting with K05 (right) for Docking Pose 3. (PDF)

S5 Fig Binding pocket (left) and the key residues interacting with K05 (right) for Docking Pose 4. (PDF)

S6 Fig 2D-Diagram of detailed interactions between K05 and Eco-MscL (Panel A), 011A and Eco-MscL (Panel B) reviewed by the best docking poses. The key of interaction types and sites is shown in Panel C.
(PDF)

S7 Fig 2D-Diagram of detailed interactions between K05 and Eco-MscL revealed by Docking Pose 3 (Panel A), and Docking Pose 4 (Panel B). The key of interaction types and sites in shown in the Panel C.
(PDF)

S8 Fig The RMSD (Root-mean-square deviation) ~ Simulation Time plot for Docking Pose 1. According to the RMSDs of nonfit ligand (the blue curve), two conformation clusters can be observed. The first cluster is from 20 to 115 ns and second from 115 to 155 ns.
(PDF)

S9 Fig A representative conformation of the first (Panels A-C) and second (Panels D-F) conformational clusters. (A) and (D): MscL/K05 in 240 POPC lipid; (B) and (E): MscL/K05 complex; (C) and (F): detailed interaction of the binding mode.
(PDF)

S10 Fig The RMSD (Root-mean-square deviation) ~ Simulation Time plot for Docking Pose 3. (PDF)

S11 Fig The RMSD (Root-mean-square deviation) ~ Simulation Time plot for Docking Pose 4. (PDF)

S12 Fig Passing-through experiment induced by an external electric field of 0.2 Volt/Å applied to DHS. DHS passed through the MscL channel 15 times within 50 nanoseconds. The distance is between the center of the DHS and the center of five LYS106 residues.
(PDF)

S13 Fig The changes of channel radii upon ligand binding. *Δr* = *r*_*MscL*/*Lig*_ − *r*_*MscL*_, where *r*_*MscL*/*Lig*_ is the channel radii for the MscL/011A or MscL/K05 complex and *r*_*MscL*_ is that for MscL protein only. The radii parameters were calculated for a set of MD snapshots from conventional MD simulations.
(PDF)

S14 Fig The changes of channel radii upon ligand binding. *Δr* = *r*_*MscL*/*Lig*_ − *r*_*MscL*_, where *r*_*MscL*/*Lig*_ is the channel radii for the MscL/011A or MscL/K05 complex and *r*_*MscL*_ is that for MscL protein only. The radii parameters were calculated for a set of channel-open conformations obtained from passing-through experiment.
(PDF)

S15 Fig The changes of channel radii upon ligand binding. *Δr* = *r*_*MscL*/*Lig*_ − *r*_*MscL*_, where *r*_*MscL*/*Lig*_ is the channel radii for the MscL/011A or MscL/K05 complex and *r*_*MscL*_ is that for MscL protein only. The radii parameters were calculated for a set of snapshots collected from conventional MD simulations for which the initial conformations are channel-open conformations.
(PDF)

S16 Fig Residues (10–40) for which channel radii were calculated are shown on a subunit structure. (PDF)

S17 Fig The RMSD (Root-mean-square deviation) ~ Simulation Time plots. MD simulations were performed starting from MscL channel-open conformations. If a threshold of 3.0 Å for RMSDs of the secondary structures (SS) is applied, 011A and K05 can maintain MscL channel-open conformations for 80 nanoseconds.
(PDF)

S1 Table List of MM-PBSA free energy components (in kcal/mol) for three top docking poses. ε_lip_ is the dielectric constant of the lipids.
(PDF)

S2 Table List of free energy components (in kcal/mol) for MM-PBSA binding free energy calculation. ε_lip_ is the dielectric constant of the lipids.
(PDF)

S3 Table Hotspot residue identification using MM-GBSA binding free energy decomposition analysis for Eco-MscL/K05. A hotspot residue is recognized when its interaction energy with the ligand is better than -1.0 kcal/mol. Those cells without numbers have neglectable interaction energies.
(PDF)

S4 Table List of radius parameters (in Å) of cycles formed by the same residues in the five chains. Note that the larger the value, the more open it is.
(PDF)
