# Supplementary material for: Novel MscL agonists that allow multiple antibiotics cytoplasmic access activate the channel through a common binding site
Source: PLoS One. 2020 Jan 24;15(1):e0228153. doi: 10.1371/journal.pone.0228153 (PMC6980572; doi:10.1371/journal.pone.0228153)
Supplement: S1 Fig — Shown are results from MJF455 cells expressing an empty vector (Null) or E. coli MscL (Eco MscL). A) The reduction of potassium was measured as the percentage of K+ remaining after overnight incubation of 70 μM K05 compared to control (non-treated with K05). Error bars reflect s.e.m. n = 9, p < .00005, unpaired TTEST. B) The reduction of glutamate was similarly measured as the percentage remaining after overnight incubation of 70 μM K05 compared to control. Error bars reflect s.e.m. n = 3, p < .01, unpaired TTEST. (PDF) [file pone.0228153.s001.pdf]

Supplemental; Small compounds modulate and bind MscL similarly

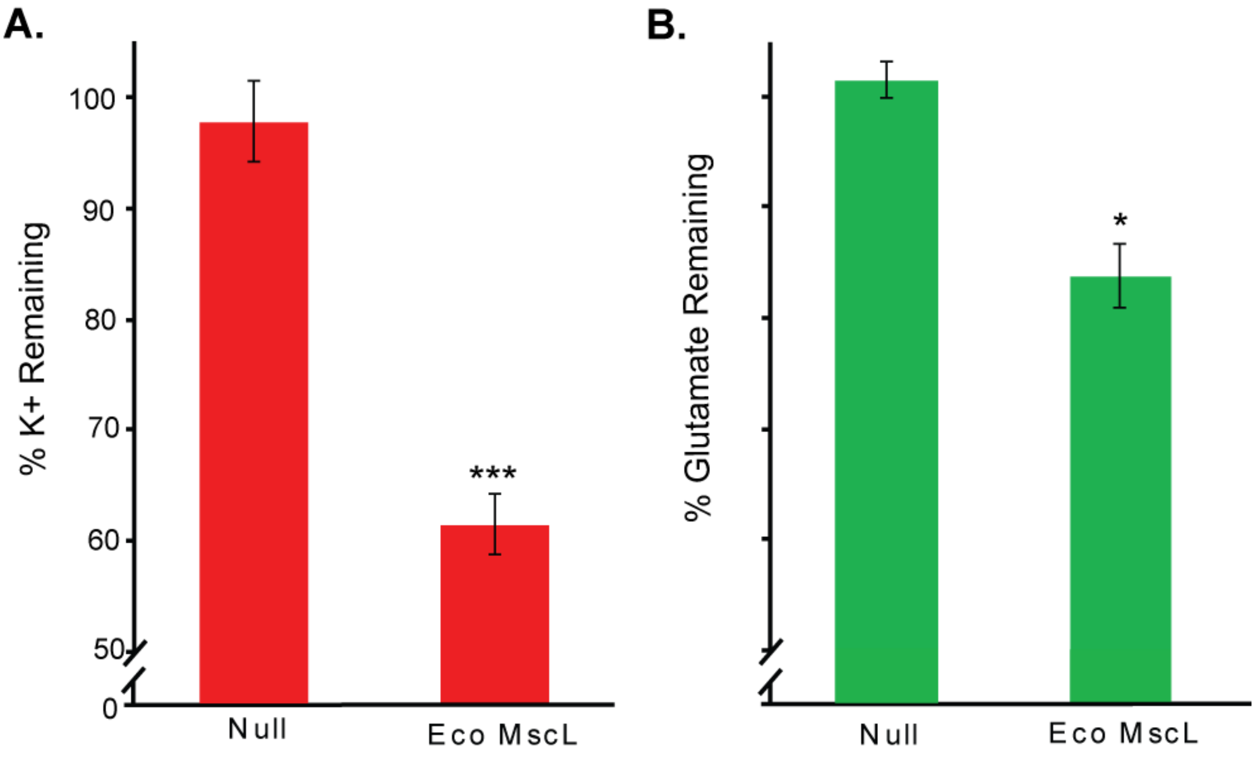

S1 Fig. K05 induces a MscL-dependent reduction in K+ and glutamate steady state. Shown are results from MJF455 cells expressing an empty vector (Null) or E. coli MscL (Eco MscL). A) The reduction of potassium was measured as the percentage of K+ remaining after overnight incubation of 70 µM K05 compared to control (non-treated with K05). Error bars reflect s.e.m. n=9, p<.00005, unpaired TTEST. B) The reduction of glutamate was similarly measured as the percentage remaining after overnight incubation of 70 µM K05 compared to control. Error bars reflect s.e.m. n=3, p<.01, unpaired TTEST.
