# Supplementary material for: Novel MscL agonists that allow multiple antibiotics cytoplasmic access activate the channel through a common binding site
Source: PLoS One. 2020 Jan 24;15(1):e0228153. doi: 10.1371/journal.pone.0228153 (PMC6980572; doi:10.1371/journal.pone.0228153)
Supplement: S2 Fig — Reduction in bacterial growth (OD600) for cultures of the E.coli MJF455 strain carrying empty plasmid (null), or expressing Eco-MscS (MscS), C. perfringens (C. perf), S. aureus (S. aur), or H. influenza (H. infl) constructs treated with compounds 011A (orange) and K05 (blue) at 60uM relative to non-treated are shown. Values represent the mean of four experiments and error bars are the SEM. (PDF) [file pone.0228153.s002.pdf]

Supplemental; Small compounds modulate and bind MscL similarly

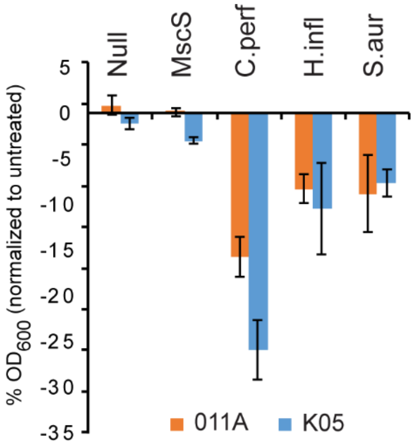

S2 Fig. MscL orthologues show a similar growth restriction profile for 011A and K05. Reduction in bacterial growth (OD_{600}) for cultures of the *E.coli* MJF455 strain carrying empty plasmid (null), or expressing Eco-MscS (MscS), *C. perfringens* (C. perf), *S. aureus* (S. aur), or *H. influenza* (H. infl) constructs treated with compounds 011A (orange) and K05 (blue) at 60uM relative to non-treated are shown. Values represent the mean of four experiments and error bars are the SEM.
