# Supplementary material for: Novel MscL agonists that allow multiple antibiotics cytoplasmic access activate the channel through a common binding site
Source: PLoS One. 2020 Jan 24;15(1):e0228153. doi: 10.1371/journal.pone.0228153 (PMC6980572; doi:10.1371/journal.pone.0228153)
Supplement: S3 Fig — The Top 1 pose (docking score = -7.22 kcal/mol) is always shown as brownish sticks and three other poses are shown in greenish sticks: Docking Pose 2 (docking score = -6.66 kcal/mol2) in Panel A, Docking Pose 3 (docking score = -6.55 kcal/mol) in Panel B and Docking Pose 4 (docking score = -6.48 kcal/mol) in Panel C. The representative conformations of Clusters 1 and 2 are shown as greenish and brownish sticks (Panel D). (PDF) [file pone.0228153.s003.pdf]

Supplemental; Small compounds modulate and bind MscL similarly

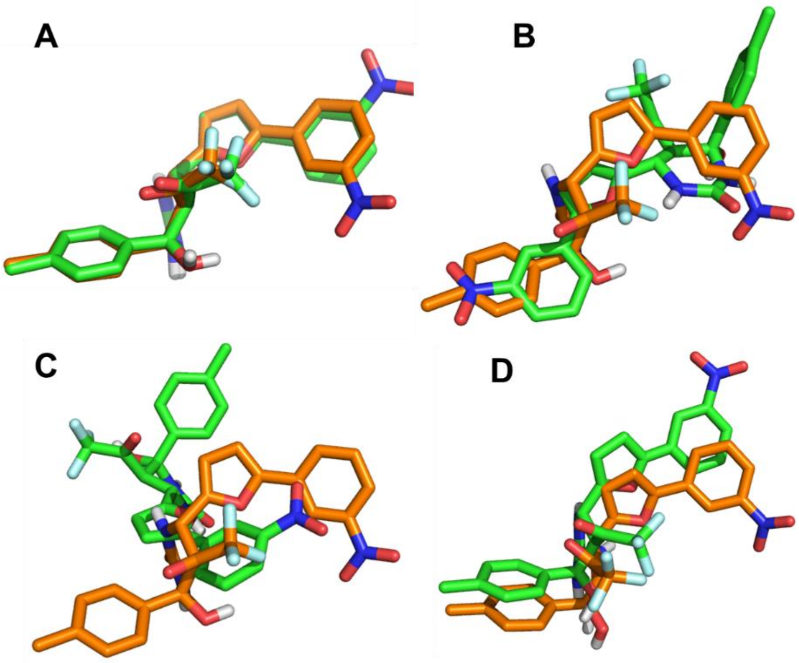

S3 Fig. Comparison between top docking poses and the representative conformations of the two clusters sampled by MD simulations. The Top 1 pose (docking score = -7.22 kcal/mol) is always shown as brownish sticks and three other poses are shown in greenish sticks: Docking Pose 2 (docking score = -6.66 kcal/mol2) in Panel A, Docking Pose 3 (docking score = -6.55 kcal/mol) in Panel B and Docking Pose 4 (docking score = -6.48 kcal/mol) in Panel C. The representative conformations of Clusters 1 and 2 are shown as greenish and brownish sticks (Panel D).
