# Supplementary material for: Novel MscL agonists that allow multiple antibiotics cytoplasmic access activate the channel through a common binding site
Source: PLoS One. 2020 Jan 24;15(1):e0228153. doi: 10.1371/journal.pone.0228153 (PMC6980572; doi:10.1371/journal.pone.0228153)
Supplement: S5 Fig — (PDF) [file pone.0228153.s005.pdf]

Supplemental; Small compounds modulate and bind MscL similarly

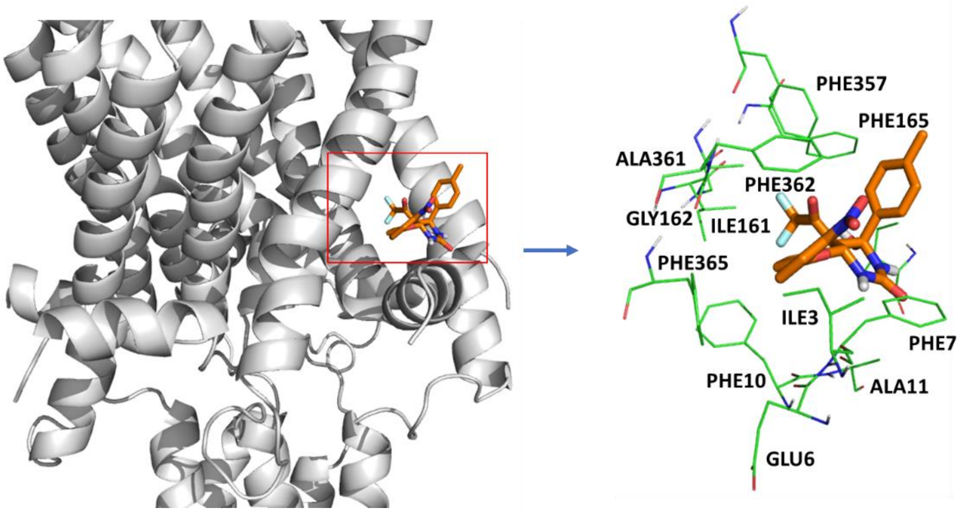

S5. Fig. Binding pocket (left) and the key residues interacting with K05 (right) for Docking Pose 4.
