# Supplementary material for: Novel MscL agonists that allow multiple antibiotics cytoplasmic access activate the channel through a common binding site
Source: PLoS One. 2020 Jan 24;15(1):e0228153. doi: 10.1371/journal.pone.0228153 (PMC6980572; doi:10.1371/journal.pone.0228153)
Supplement: S7 Fig — The key of interaction types and sites in shown in the Panel C. (PDF) [file pone.0228153.s007.pdf]

Supplemental; Small compounds modulate and bind MscL similarly

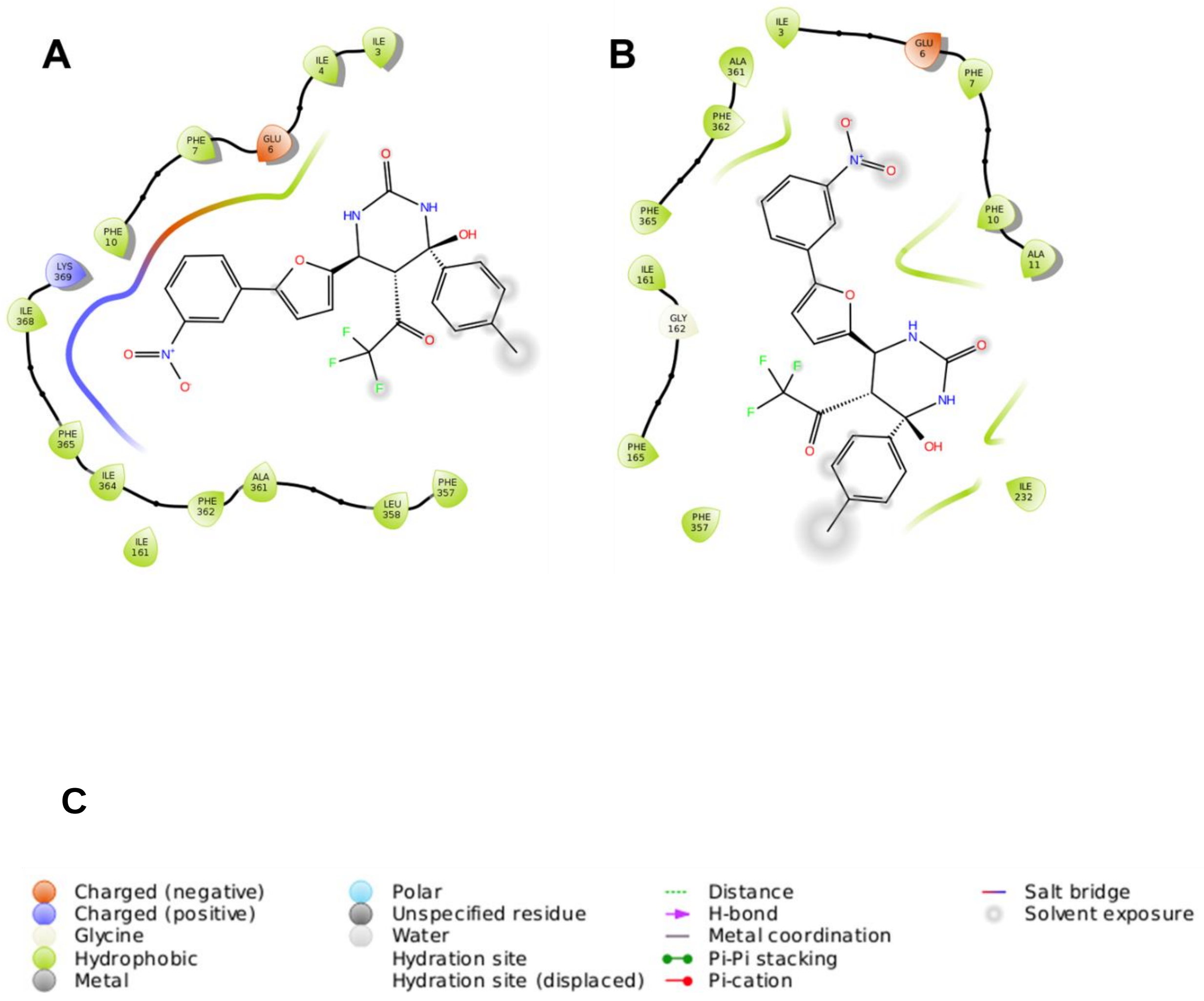

S7 Fig. **2D-Diagram of detailed interactions between K05 and Eco-MscL revealed by Docking Pose 3 (Panel A), and Docking Pose 4 (Panel B).** The key of interaction types and sites in shown in the Panel C.
