# Supplementary material for: Novel MscL agonists that allow multiple antibiotics cytoplasmic access activate the channel through a common binding site
Source: PLoS One. 2020 Jan 24;15(1):e0228153. doi: 10.1371/journal.pone.0228153 (PMC6980572; doi:10.1371/journal.pone.0228153)
Supplement: S8 Fig — According to the RMSDs of nonfit ligand (the blue curve), two conformation clusters can be observed. The first cluster is from 20 to 115 ns and second from 115 to 155 ns. (PDF) [file pone.0228153.s008.pdf]

Supplemental; Small compounds modulate and bind MscL similarly

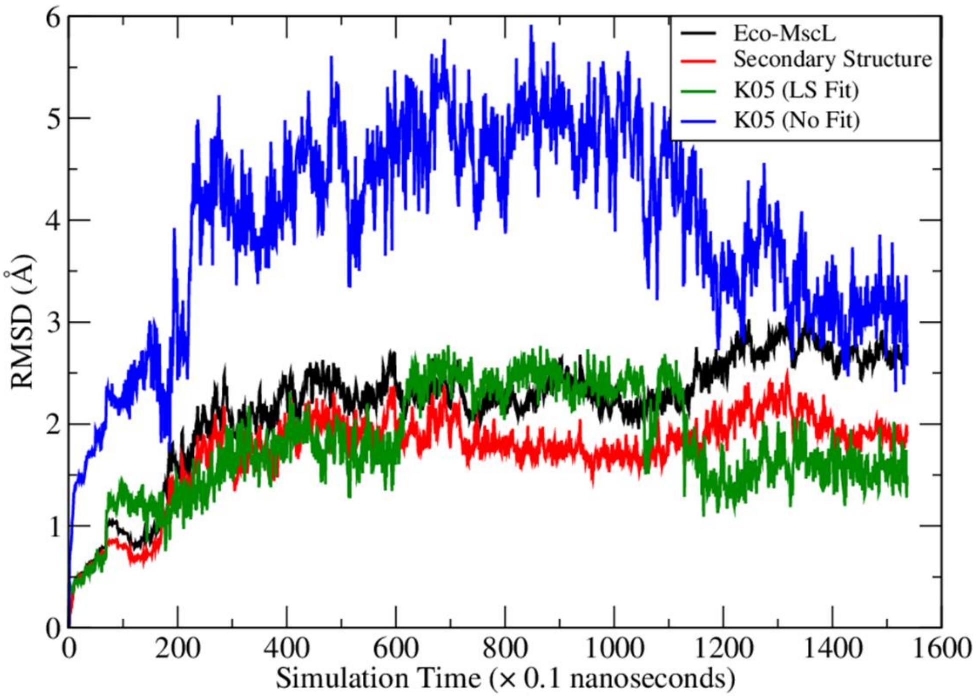

S8 Fig. The RMSD (Root-mean-square deviation) ~ Simulation Time plot for Docking Pose 1. According to the RMSDs of nonfit ligand (the blue curve), two conformation clusters can be observed. The first cluster is from 20 to 115 ns and second from 115 to 155 ns.
