# Supplementary material for: Novel MscL agonists that allow multiple antibiotics cytoplasmic access activate the channel through a common binding site
Source: PLoS One. 2020 Jan 24;15(1):e0228153. doi: 10.1371/journal.pone.0228153 (PMC6980572; doi:10.1371/journal.pone.0228153)
Supplement: S9 Fig — (A) and (D): MscL/K05 in 240 POPC lipid; (B) and (E): MscL/K05 complex; (C) and (F): detailed interaction of the binding mode. (PDF) [file pone.0228153.s009.pdf]

Supplemental; Small compounds modulate and bind MscL similarly

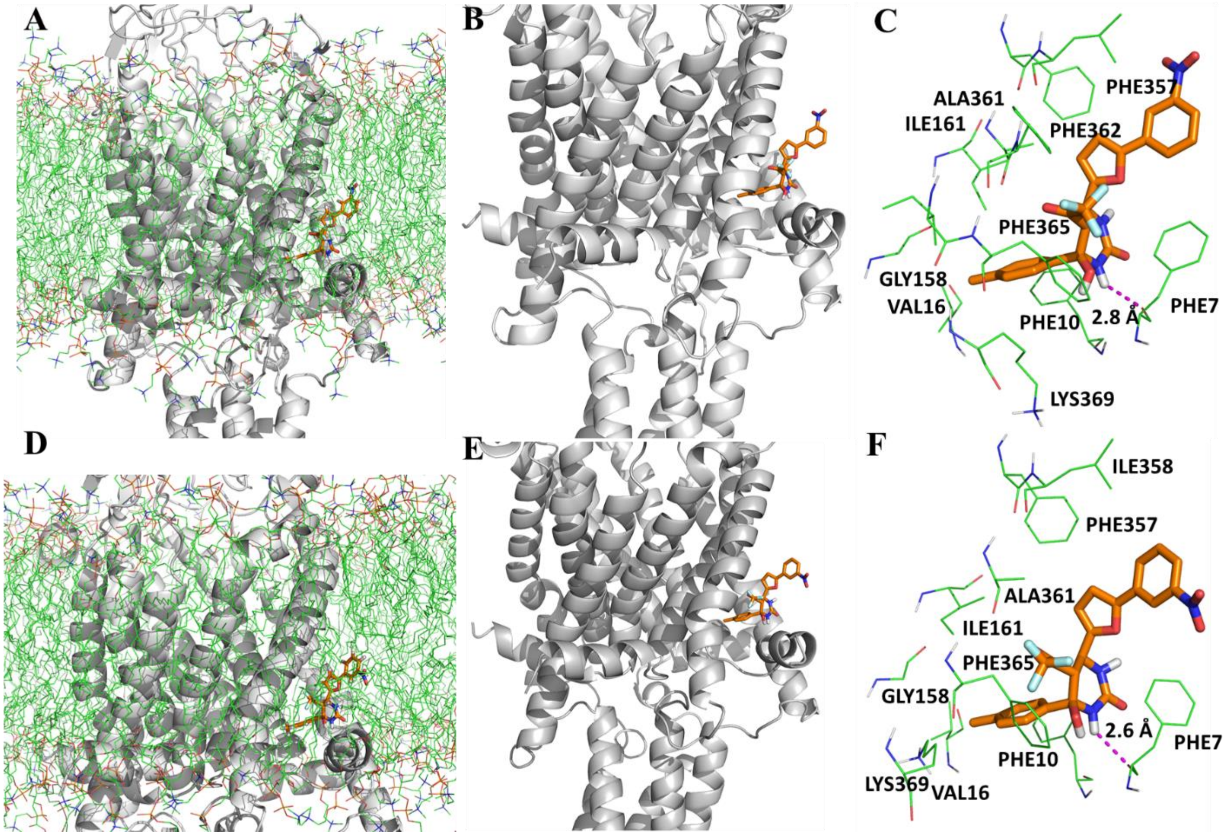

S9 Fig. A representative conformation of the first (Panels A-C) and second (Panels D-F) conformational clusters. (A) and (D): MscL/K05 in 240 POPC lipid; (B) and (E): MscL/K05 complex; (C) and (F): detailed interaction of the binding mode.
