# Supplementary material for: Novel MscL agonists that allow multiple antibiotics cytoplasmic access activate the channel through a common binding site
Source: PLoS One. 2020 Jan 24;15(1):e0228153. doi: 10.1371/journal.pone.0228153 (PMC6980572; doi:10.1371/journal.pone.0228153)
Supplement: S10 Fig — (PDF) [file pone.0228153.s010.pdf]

**Supplemental; Small compounds modulate and bind MscL similarly**

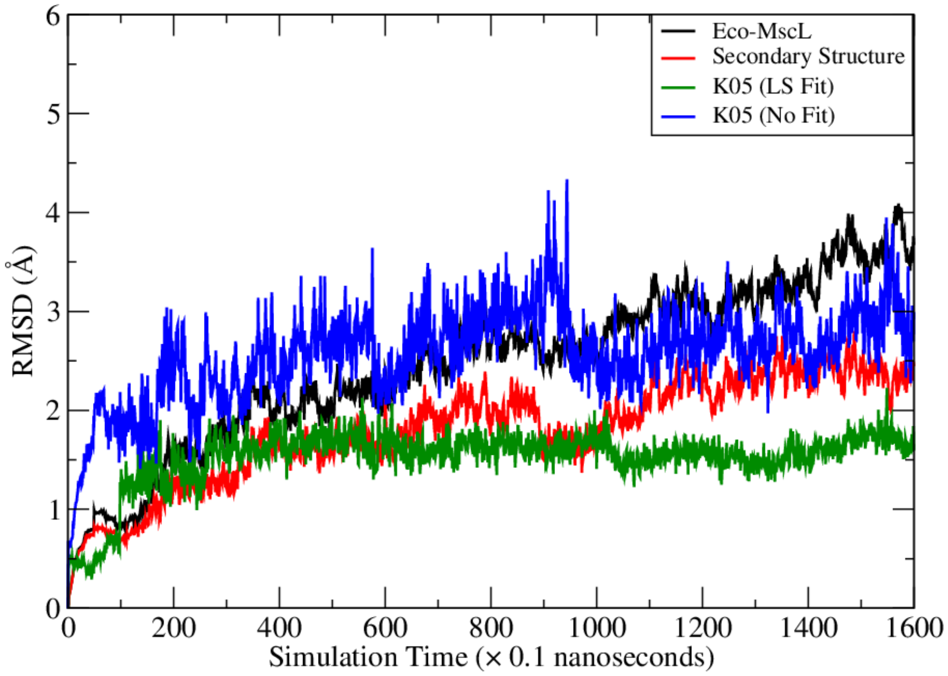


**S10 Fig. The RMSD (Root-mean-square deviation) ~ Simulation Time plot for Docking Pose 3.**
