# Supplementary material for: Novel MscL agonists that allow multiple antibiotics cytoplasmic access activate the channel through a common binding site
Source: PLoS One. 2020 Jan 24;15(1):e0228153. doi: 10.1371/journal.pone.0228153 (PMC6980572; doi:10.1371/journal.pone.0228153)
Supplement: S11 Fig — (PDF) [file pone.0228153.s011.pdf]

Supplemental; Small compounds modulate and bind MscL similarly

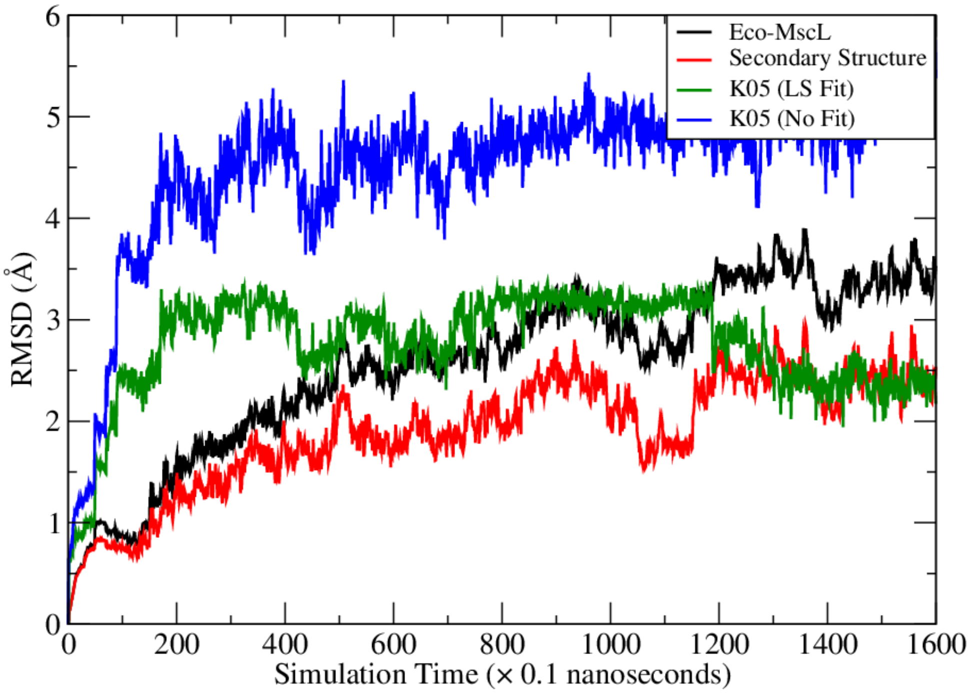

S11 Fig. The RMSD (Root-mean-square deviation) ~ Simulation Time plot for Docking Pose 4.
