# Supplementary material for: Novel MscL agonists that allow multiple antibiotics cytoplasmic access activate the channel through a common binding site
Source: PLoS One. 2020 Jan 24;15(1):e0228153. doi: 10.1371/journal.pone.0228153 (PMC6980572; doi:10.1371/journal.pone.0228153)
Supplement: S12 Fig — DHS passed through the MscL channel 15 times within 50 nanoseconds. The distance is between the center of the DHS and the center of five LYS106 residues. (PDF) [file pone.0228153.s012.pdf]

Supplemental; Small compounds modulate and bind MscL similarly

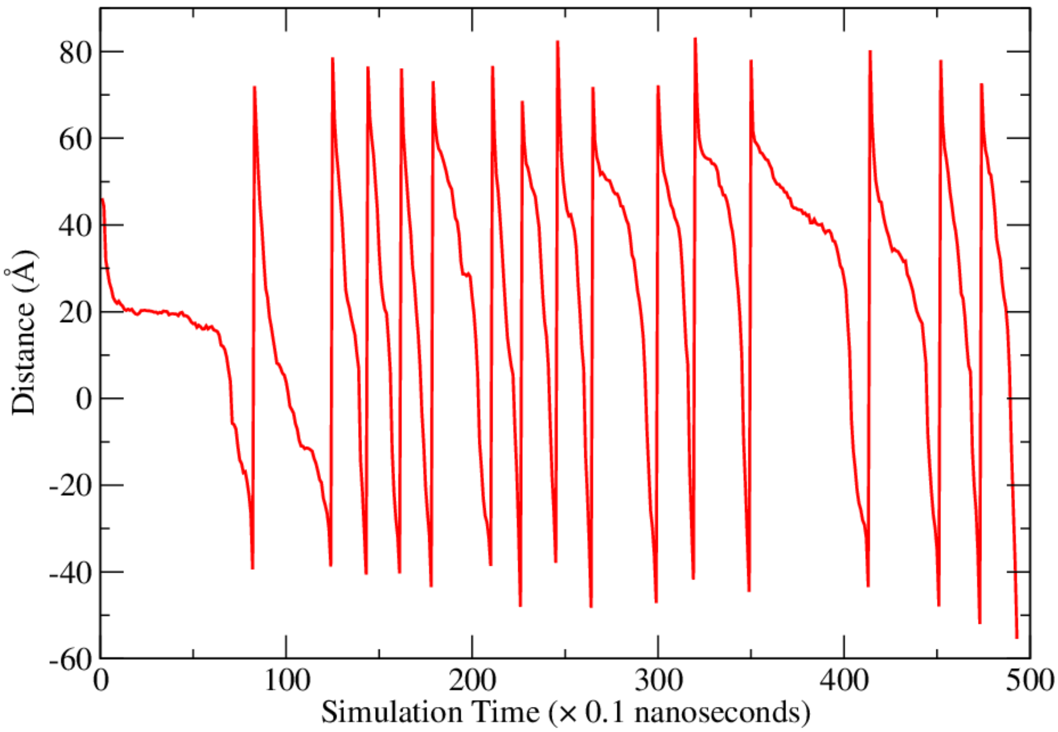

S12 Fig. Passing-through experiment induced by an external electric field of 0.2 Volt/Å applied to DHS. DHS passed through the MscL channel 15 times within 50 nanoseconds. The distance is between the center of the DHS and the center of five LYS106 residues.
