# Supplementary material for: Novel MscL agonists that allow multiple antibiotics cytoplasmic access activate the channel through a common binding site
Source: PLoS One. 2020 Jan 24;15(1):e0228153. doi: 10.1371/journal.pone.0228153 (PMC6980572; doi:10.1371/journal.pone.0228153)
Supplement: S14 Fig — The radii parameters were calculated for a set of channel-open conformations obtained from passing-through experiment. (PDF) [file pone.0228153.s014.pdf]

Supplemental; Small compounds modulate and bind MscL similarly

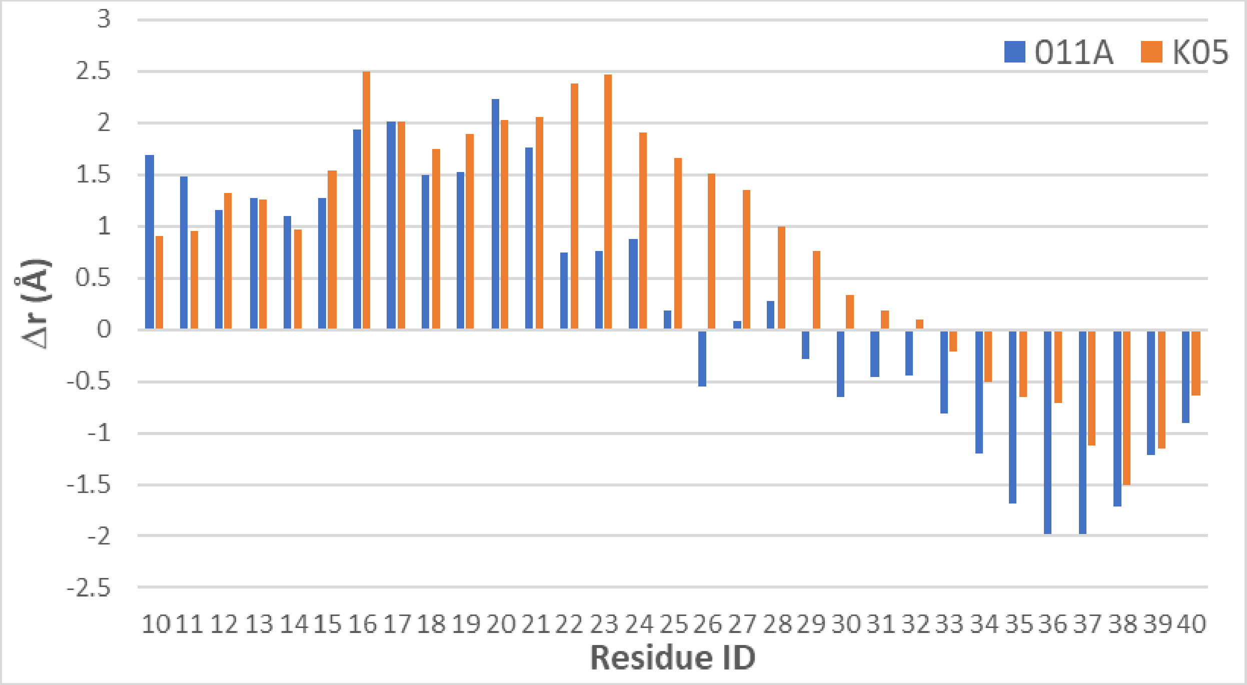

S14 Fig. The changes of channel radii upon ligand binding. $\Delta r = r_{MscL/Lig} - r_{MscL}$**, where** $r_{MscL/Lig}$ **is the channel radii for the MscL/011A or MscL/K05 complex and** r_{MscL} **is that for MscL protein only.** The radii parameters were calculated for a set of channel-open conformations obtained from passing-through experiment.
