# Supplementary material for: Novel MscL agonists that allow multiple antibiotics cytoplasmic access activate the channel through a common binding site
Source: PLoS One. 2020 Jan 24;15(1):e0228153. doi: 10.1371/journal.pone.0228153 (PMC6980572; doi:10.1371/journal.pone.0228153)
Supplement: S16 Fig — (PDF) [file pone.0228153.s016.pdf]

Supplemental; Small compounds modulate and bind MscL similarly

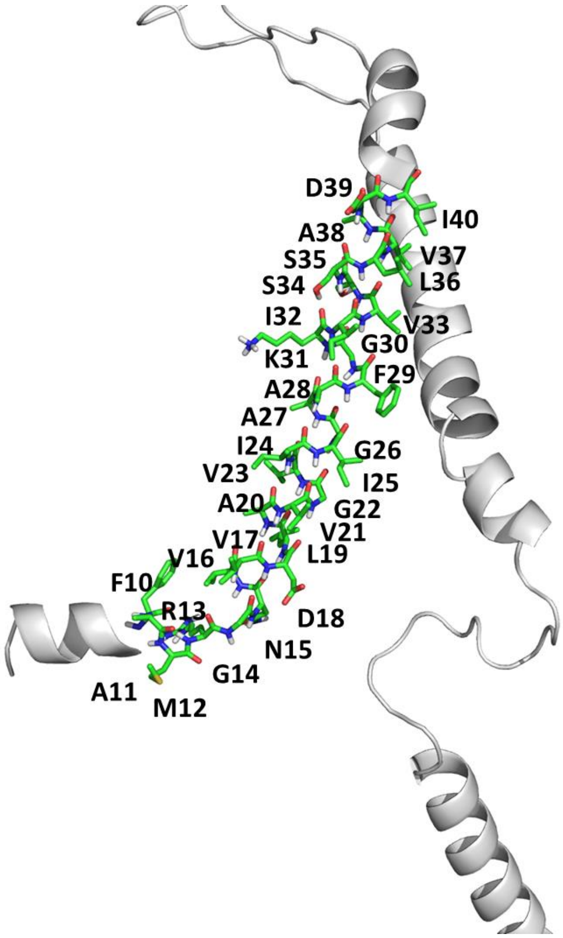

S16 Fig. Residues (10-40) for which channel radii were calculated are shown on a subunit structure.

Supplemental; Small compounds modulate and bind MscL similarly

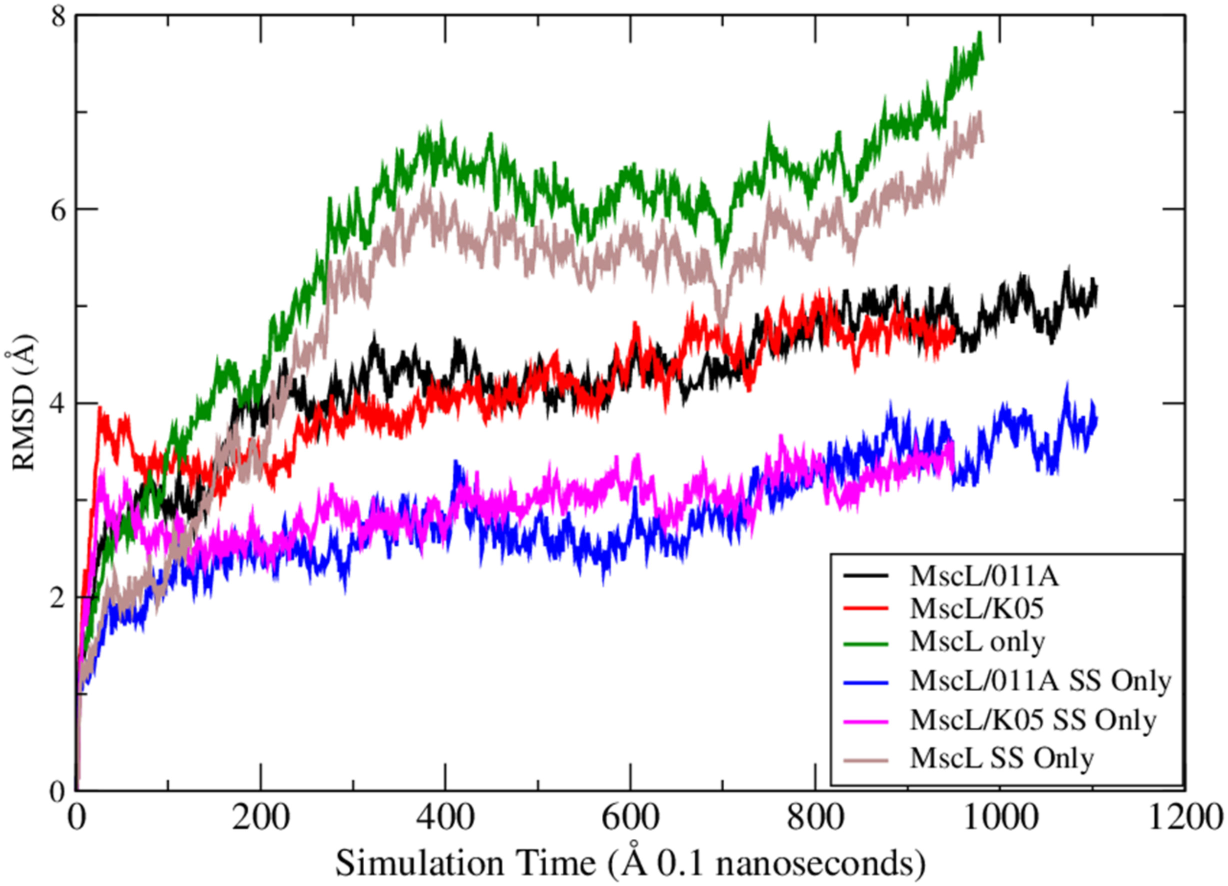

S17 Fig. The RMSD (Root-mean-square deviation) ~ Simulation Time plots. MD simulations were performed starting from MscL channel-open conformations. If a threshold of 3.0 Å for RMSDs of the secondary structures (SS) is applied, 011A and K05 can maintain MscL channel-open conformations for 80 nanoseconds.

Supplemental; Small compounds modulate and bind MscL similarly

S1Table. List of MM-PBSA free energy components (in kcal/mol) for three top docking poses. ε_{lip} is the dielectric constant of the lipids.

System	vdW	EEL	ΔG_{pol}^{PB}			$\Delta G_{nonpolar}^{SAS}$	TΔs	MM-PBSA		
			ε_{lip}=1	ε_{lip}=2	ε_{lip}=4			ε_{lip}=1	ε_{lip}=2	ε_{lip}=4
Docking Pose 1	-5128.70 ± 1.97	-46350.91 ± 5.20	-5467.61 ± 2.70	-5028.58 ± 1.10	-4786.99 ± 3.34	204.30 ± 0.06	7750.53 ± 0.57	-64493.46 ± 7.08	-64054.43 ± 5.23	-63812.83 ± 6.16
Docking Pose 3	-5108.47 ± 4.26	-46619.83 ± 21.81	-5114.58 ± 29.76	-4675.69 ± 28.24	-4426.39 ± 27.68	206.34 ± 0.10	7761.97 ± 0.46	-64398.50 ± 20.81	-63959.62 ± 22.02	-63710.32 ± 22.77
Docking Pose 4	-5112.89 ± 6.02	-46744.48 ± 29.90	-4955.35 ± 7.02	-4536.67 ± 8.16	-4303.64 ± 9.45	204.70 ± 0.03	7756.02 ± 0.73	-64364.04 ± 23.37	-63945.35 ± 22.06	-63712.32 ± 22.90

S2 Table. List of free energy components (in kcal/mol) for MM-PBSA binding free energy calculation. ε_{lip} is the dielectric constant of the lipids.

System	vdW	EEL	ΔG_{pol}^{PB}			$\Delta G_{nonpolar}^{SAS}$	TΔs	Binding		
			ε_{lip}=1	ε_{lip}=2	ε_{lip}=4			ε_{lip}=1	ε_{lip}=2	ε_{lip}=4
Pose 1 Cluster 1	-35.36 ± 0.74	-3.73 ± 0.34	16.31 ± 0.30	15.37 ± 0.28	15.06 ± 0.23	-3.67 ± 0.03	-19.61 ± 0.13	-6.86 ± 0.76	-7.79 ± 0.75	-8.10 ± 0.70
Pose 1 Cluster 2	-33.91 ± 0.71	-7.03 ± 0.46	19.53 ± 0.39	17.34 ± 0.23	16.39 ± 0.29	-3.59 ± 0.03	-19.14 ± 0.28	-5.76 ± 0.58	-8.06 ± 0.55	-9.01 ± 0.60
Pose 1 Combined	-34.94 ± 0.73	-4.69 ± 0.13	17.25 ± 0.16	15.93 ± 0.14	15.44 ± 0.12	-3.65 ± 0.03	-19.47 ± 0.17	-6.55± 0.66	-7.90 ± 0.62	-8.37± 0.57
Pose 3	-45.64 ± 0.17	-10.86 ± 0.36	18.11 ± 0.13	16.96 ± 0.02	16.69 ± 0.03	-3.61 ± 0.02	-21.37 ± 0.07	-20.62± 0.24	-21.78 ± 0.23	-22.05± 0.26
Pose 4	-33.55 ± 0.09	-8.18 ± 0.19	21.10 ± 0.13	17.64 ± 0.21	16.37 ± 0.26	-3.49 ± 0.01	-19.39 ± 0.04	-4.73± 0.20	-8.20 ± 0.17	-9.47± 0.20

S3 Table. Hotspot residue identification using MM-GBSA binding free energy decomposition analysis for Eco-MscL/K05. A hotspot residue is recognized when its interaction energy with the ligand is better than -1.0 kcal/mol. Those cells without numbers have neglectable interaction energies.

Hotspot Residue	Scenario 3 Channel-Close	Scenario 7 Channel-Open
PHE7	-6.58	
PHE10	-5.12	-0.11
ALA11	-0.27	-1.25

Supplemental; Small compounds modulate and bind MscL similarly

ARG13		-3.93
**GLY14**		**-3.98**
**ASN15**		**-4.01**
**VAL17**		**-2.18**
**ASP18**		**-4.08**
PRO115		-1.01
ILE161/25	-2.99	>-0.1
ASN236		-3.09
ASN239		-1.93
ARG240		-6.84
PRO249		-2.88
PRO251		-3.51
PHE357/85	-2.44	
PHE358/86	-2.19	
PHE361/89	-2.00	
PHE365/93	-2.46	
PHE554		-1.03
ARG557		-2.36

Supplemental; Small compounds modulate and bind MscL similarly

S4 Table. List of radius parameters (in Å) of cycles formed by the same residues in the five chains. Note that the larger the value, the more open it is.

Residue	**Scenario 1 MscL only[1]**	**Scenario 2 MscL/011[2]**	**Scenario 3 MscL/K05[3]**	**Scenario 4 MscL/011[4]**	**Scenario 5 MscL/K05[5]**	**Scenario 6 MscL/011[6]**	**Scenario 7 MscL/K05[7]**
PHE10	16.42 ± 0.18	16.04 ± 0.22	16.56 ± 0.19	18.11 ± 0.16	17.33 ± 0.46	17.56 ± 0.26	16.50 ± 0.25
ALA11	16.89 ± 0.24	16.36 ± 0.20	17.14 ± 0.30	18.37 ± 0.22	17.84 ± 0.57	17.87 ± 0.29	17.24 ± 0.31
MET12	16.55 ± 0.22	16.02 ± 0.19	16.83 ± 0.40	17.70 ± 0.40	17.86 ± 0.74	16.91 ± 0.31	16.82 ± 0.38
ARG13	13.69 ± 0.20	13.13 ± 0.20	13.89 ± 0.36	14.97 ± 0.43	14.95 ± 0.72	14.15 ± 0.33	13.82 ± 0.39
GLY14	11.96 ± 0.22	11.18 ± 0.23	11.79 ± 0.34	13.05 ± 0.67	12.92 ± 0.74	12.75 ± 0.35	11.63 ± 0.41
**ASN15**	**8.96 ± 0.20**	**8.25 ± 0.20**	**8.69 ± 0.28**	**10.24 ± 0.55**	**10.50 ± 0.81**	**10.15 ± 0.33**	**8.90 ± 0.45**
**VAL16**	**8.57 ± 0.15**	**7.74 ± 0.23**	**8.21 ± 0.18**	**10.51 ± 0.48**	**11.06 ± 0.61**	**10.24 ± 0.26**	**9.85 ± 0.33**
**VAL17**	**10.14 ± 0.12**	**9.64 ± 0.20**	**9.74 ± 0.21**	**12.15 ± 0.36**	**12.15 ± 0.45**	**11.69 ± 0.27**	**11.29 ± 0.33**
**ASP18**	**9.38 ± 0.13**	**9.00 ± 0.17**	**8.96 ± 0.23**	**10.88 ± 0.25**	**11.13 ± 0.66**	**10.34 ± 0.27**	**10.30 ± 0.30**
**LEU19**	**6.84 ± 0.13**	**6.39 ± 0.21**	**6.57 ± 0.20**	**8.36 ± 0.28**	**8.73 ± 0.64**	**7.89 ± 0.26**	**8.02 ± 0.21**
**ALA20**	**7.47 ± 0.12**	**7.24 ± 0.20**	**7.37 ± 0.18**	**9.70 ± 0.22**	**9.50 ± 0.54**	**9.18 ± 0.31**	**9.17 ± 0.21**
**VAL21**	**9.31 ± 0.13**	**9.37 ± 0.13**	**9.12 ± 0.14**	**11.07 ± 0.25**	**11.36 ± 0.56**	**10.54 ± 0.22**	**10.99 ± 0.20**
**GLY22**	**8.19 ± 0.14**	**8.31 ± 0.15**	**8.09 ± 0.14**	**8.94 ± 0.26**	**10.57 ± 0.43**	**8.60 ± 0.19**	**10.35 ± 0.19**
**VAL23**	**6.63 ± 0.13**	**6.56 ± 0.17**	**6.50 ± 0.14**	**7.39 ± 0.13**	**9.10 ± 0.34**	**7.02 ± 0.18**	**9.34 ± 0.17**
**ILE24**	**8.84 ± 0.13**	**8.71 ± 0.15**	**8.63 ± 0.15**	**9.71 ± 0.09**	**10.75 ± 0.32**	**9.38 ± 0.20**	**10.90 ± 0.19**
ILE25	10.86 ± 0.12	10.97 ± 0.15	10.73 ± 0.16	11.04 ± 0.13	12.51 ± 0.33	10.86 ± 0.15	12.60 ± 0.21
GLY26	9.62 ± 0.18	9.90 ± 0.22	9.54 ± 0.25	9.07 ± 0.21	11.13 ± 0.35	9.04 ± 0.22	11.49 ± 0.28
ALA27	8.75 ± 0.16	8.91 ± 0.20	8.64 ± 0.22	8.83 ± 0.20	10.09 ± 0.45	8.65 ± 0.16	10.60 ± 0.30
ALA28	11.56 ± 0.16	11.65 ± 0.18	11.42 ± 0.22	11.83 ± 0.17	12.56 ± 0.40	11.63 ± 0.18	12.85 ± 0.28
PHE29	13.23 ± 0.15	13.44 ± 0.20	13.06 ± 0.24	12.94 ± 0.15	13.99 ± 0.49	12.92 ± 0.14	14.25 ± 0.23
GLY30	11.89 ± 0.17	12.17 ± 0.23	11.65 ± 0.26	11.24 ± 0.20	12.22 ± 0.56	11.33 ± 0.16	12.58 ± 0.24
LYS31	12.19 ± 0.19	12.31 ± 0.21	12.01 ± 0.24	11.72 ± 0.23	12.37 ± 0.43	11.75 ± 0.23	12.57 ± 0.22
ILE32	15.29 ± 0.18	15.40 ± 0.21	15.12 ± 0.24	14.84 ± 0.22	15.38 ± 0.41	14.84 ± 0.23	15.58 ± 0.21
VAL33	15.85 ± 0.15	16.16 ± 0.25	15.80 ± 0.31	15.03 ± 0.24	15.63 ± 0.52	15.14 ± 0.18	16.02 ± 0.20
SER34	14.18 ± 0.18	14.47 ± 0.29	14.01 ± 0.34	12.98 ± 0.20	13.67 ± 0.52	13.22 ± 0.20	13.99 ± 0.19
SER35	15.59 ± 0.26	15.66 ± 0.24	15.17 ± 0.30	13.90 ± 0.16	14.93 ± 0.48	14.07 ± 0.23	15.00 ± 0.18
LEU36	18.42 ± 0.20	18.59 ± 0.25	18.11 ± 0.30	16.44 ± 0.21	17.71 ± 0.45	16.84 ± 0.22	17.85 ± 0.17
VAL37	17.95 ± 0.19	18.22 ± 0.31	17.70 ± 0.32	15.97 ± 0.16	16.82 ± 0.46	16.47 ± 0.21	17.12 ± 0.18
ALA38	16.05 ± 0.23	16.27 ± 0.32	15.73 ± 0.32	14.34 ± 0.24	14.54 ± 0.51	14.68 ± 0.23	14.80 ± 0.24
ASP39	17.48 ± 0.27	17.52 ± 0.29	17.09 ± 0.31	16.27 ± 0.26	16.32 ± 0.54	16.61 ± 0.23	16.40 ± 0.28
ILE40	20.09 ± 0.25	20.04 ± 0.26	19.69 ± 0.30	19.18 ± 0.22	19.45 ± 0.54	19.56 ± 0.23	19.62 ± 0.27

1. MscL only, 1222 snapshots, conventional MD
2. MscL/011A, 1717 snapshots, conventional MD
3. MscL/K05, 1537 snapshots, conventional MD
4. MscL/011A, 10 channel-open conformations collected from the “passing-through” experiment
5. MscL/K05, 23 channel-open conformations collected from the “passing-through” experiment
6. MscL/011A, 204 snapshots, conventional MD from a representative active conformation
7. MscL/K05, 604 snapshots, conventional MD from a representative active conformation
