# Supplementary material for: Novel MscL agonists that allow multiple antibiotics cytoplasmic access activate the channel through a common binding site
Source: PLoS One. 2020 Jan 24;15(1):e0228153. doi: 10.1371/journal.pone.0228153 (PMC6980572; doi:10.1371/journal.pone.0228153)
Supplement: S17 Fig — If a threshold of 3.0 Å for RMSDs of the secondary structures (SS) is applied, 011A and K05 can maintain MscL channel-open conformations for 80 nanoseconds. (PDF) [file pone.0228153.s017.pdf]

Supplemental; Small compounds modulate and bind MscL similarly

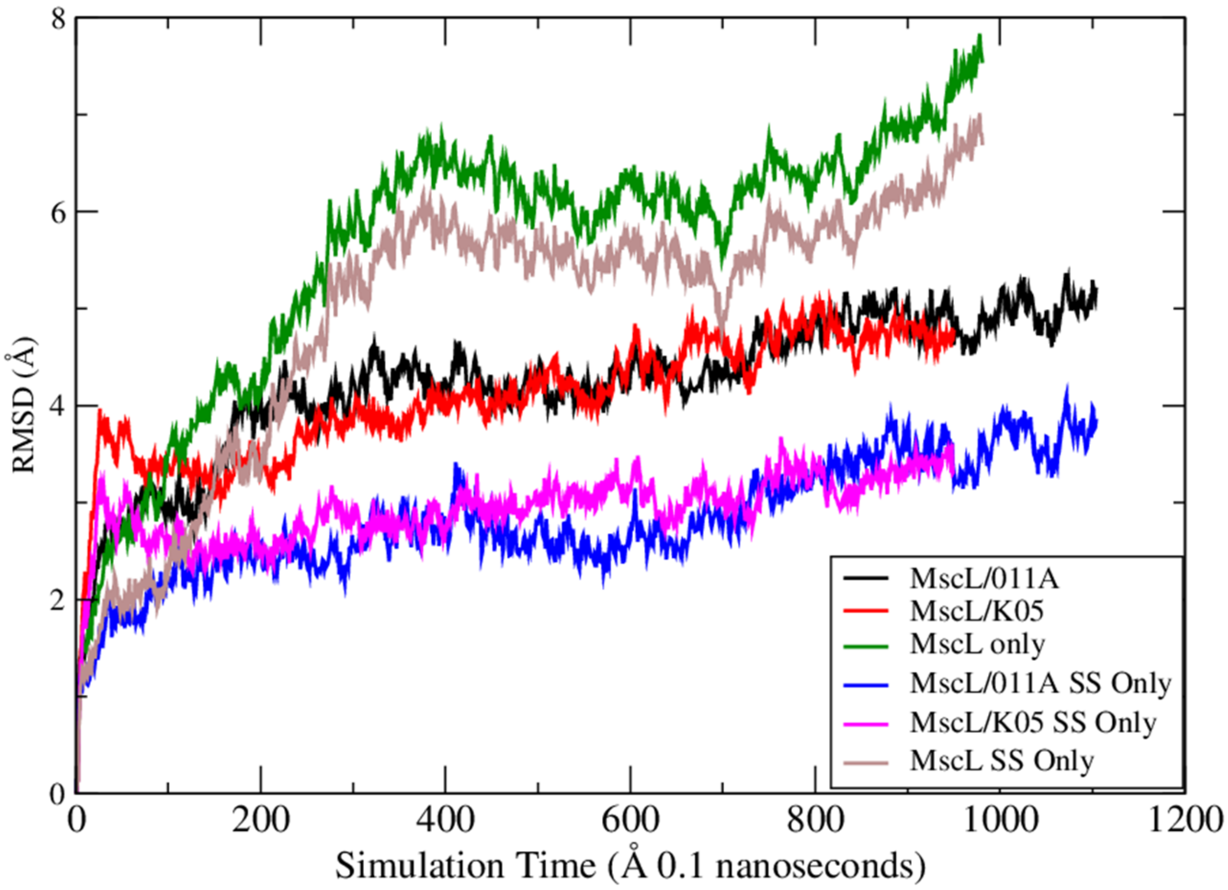

S17 Fig. The RMSD (Root-mean-square deviation) ~ Simulation Time plots. MD simulations were performed starting from MscL channel-open conformations. If a threshold of 3.0 Å for RMSDs of the secondary structures (SS) is applied, 011A and K05 can maintain MscL channel-open conformations for 80 nanoseconds.
