# Supplementary material for: Novel MscL agonists that allow multiple antibiotics cytoplasmic access activate the channel through a common binding site
Source: PLoS One. 2020 Jan 24;15(1):e0228153. doi: 10.1371/journal.pone.0228153 (PMC6980572; doi:10.1371/journal.pone.0228153)
Supplement: S1 Table — εlip is the dielectric constant of the lipids. (PDF) [file pone.0228153.s018.pdf]

Supplemental; Small compounds modulate and bind MscL similarly

S1Table. List of MM-PBSA free energy components (in kcal/mol) for three top docking poses. ε_{lip} is the dielectric constant of the lipids.

System	vdW	EEL	ΔG_{pol}^{PB}			$\Delta G_{nonpolar}^{SAS}$	TΔs	MM-PBSA		
			ε_{lip}=1	ε_{lip}=2	ε_{lip}=4			ε_{lip}=1	ε_{lip}=2	ε_{lip}=4
Docking Pose 1	-5128.70 ± 1.97	-46350.91 ± 5.20	-5467.61 ± 2.70	-5028.58 ± 1.10	-4786.99 ± 3.34	204.30 ± 0.06	7750.53 ± 0.57	-64493.46 ± 7.08	-64054.43 ± 5.23	-63812.83 ± 6.16
Docking Pose 3	-5108.47 ± 4.26	-46619.83 ± 21.81	-5114.58 ± 29.76	-4675.69 ± 28.24	-4426.39 ± 27.68	206.34 ± 0.10	7761.97 ± 0.46	-64398.50 ± 20.81	-63959.62 ± 22.02	-63710.32 ± 22.77
Docking Pose 4	-5112.89 ± 6.02	-46744.48 ± 29.90	-4955.35 ± 7.02	-4536.67 ± 8.16	-4303.64 ± 9.45	204.70 ± 0.03	7756.02 ± 0.73	-64364.04 ± 23.37	-63945.35 ± 22.06	-63712.32 ± 22.90
