# Supplementary material for: Novel MscL agonists that allow multiple antibiotics cytoplasmic access activate the channel through a common binding site
Source: PLoS One. 2020 Jan 24;15(1):e0228153. doi: 10.1371/journal.pone.0228153 (PMC6980572; doi:10.1371/journal.pone.0228153)
Supplement: S2 Table — εlip is the dielectric constant of the lipids. (PDF) [file pone.0228153.s019.pdf]

Supplemental; Small compounds modulate and bind MscL similarly

S2 Table. List of free energy components (in kcal/mol) for MM-PBSA binding free energy calculation. ε_{lip} is the dielectric constant of the lipids.

System	vdW	EEL	ΔG_{pol}^{PB}			$\Delta G_{nonpolar}^{SAS}$	TΔs	Binding		
			$\varepsilon_{lip}=1$	$\varepsilon_{lip}=2$	$\varepsilon_{lip}=4$			$\varepsilon_{lip}=1$	$\varepsilon_{lip}=2$	$\varepsilon_{lip}=4$
Pose 1 Cluster 1	-35.36 ± 0.74	-3.73 ± 0.34	16.31 ± 0.30	15.37 ± 0.28	15.06 ± 0.23	-3.67 ± 0.03	-19.61 ± 0.13	-6.86 ± 0.76	-7.79 ± 0.75	-8.10 ± 0.70
Pose 1 Cluster 2	-33.91 ± 0.71	-7.03 ± 0.46	19.53 ± 0.39	17.34 ± 0.23	16.39 ± 0.29	-3.59 ± 0.03	-19.14 ± 0.28	-5.76 ± 0.58	-8.06 ± 0.55	-9.01 ± 0.60
Pose 1 Combined	-34.94 ± 0.73	-4.69 ± 0.13	17.25 ± 0.16	15.93 ± 0.14	15.44 ± 0.12	-3.65 ± 0.03	-19.47 ± 0.17	-6.55± 0.66	-7.90 ± 0.62	-8.37± 0.57
Pose 3	-45.64 ± 0.17	-10.86 ± 0.36	18.11 ± 0.13	16.96 ± 0.02	16.69 ± 0.03	-3.61 ± 0.02	-21.37 ± 0.07	-20.62± 0.24	-21.78 ± 0.23	-22.05± 0.26
Pose 4	-33.55 ± 0.09	-8.18 ± 0.19	21.10 ± 0.13	17.64 ± 0.21	16.37 ± 0.26	-3.49 ± 0.01	-19.39 ± 0.04	-4.73± 0.20	-8.20 ± 0.17	-9.47± 0.20
