# Supplementary material for: Novel MscL agonists that allow multiple antibiotics cytoplasmic access activate the channel through a common binding site
Source: PLoS One. 2020 Jan 24;15(1):e0228153. doi: 10.1371/journal.pone.0228153 (PMC6980572; doi:10.1371/journal.pone.0228153)
Supplement: S3 Table — A hotspot residue is recognized when its interaction energy with the ligand is better than -1.0 kcal/mol. Those cells without numbers have neglectable interaction energies. (PDF) [file pone.0228153.s020.pdf]

Supplemental; Small compounds modulate and bind MscL similarly

S3 Table. Hotspot residue identification using MM-GBSA binding free energy decomposition analysis for Eco-MscL/K05. A hotspot residue is recognized when its interaction energy with the ligand is better than -1.0 kcal/mol. Those cells without numbers have neglectable interaction energies.

**Hotspot Residue**	**Scenario 3 Channel-Close**	**Scenario 7 Channel-Open**
PHE7	-6.58	
PHE10	-5.12	-0.11
ALA11	-0.27	-1.25
ARG13		-3.93
**GLY14**		**-3.98**
**ASN15**		**-4.01**
**VAL17**		**-2.18**
**ASP18**		**-4.08**
PRO115		-1.01
ILE161/25	-2.99	>-0.1
ASN236		-3.09
ASN239		-1.93
ARG240		-6.84
PRO249		-2.88
PRO251		-3.51
PHE357/85	-2.44	
PHE358/86	-2.19	
PHE361/89	-2.00	
PHE365/93	-2.46	
PHE554		-1.03
ARG557		-2.36
