# Supplementary material for: Novel MscL agonists that allow multiple antibiotics cytoplasmic access activate the channel through a common binding site
Source: PLoS One. 2020 Jan 24;15(1):e0228153. doi: 10.1371/journal.pone.0228153 (PMC6980572; doi:10.1371/journal.pone.0228153)
Supplement: S4 Table — Note that the larger the value, the more open it is. (PDF) [file pone.0228153.s021.pdf]

Supplemental; Small compounds modulate and bind MscL similarly

S4 Table. List of radius parameters (in Å) of cycles formed by the same residues in the five chains. Note that the larger the value, the more open it is.

Residue	**Scenario 1 MscL only[1]**	**Scenario 2 MscL/011[2]**	**Scenario 3 MscL/K05[3]**	**Scenario 4 MscL/011[4]**	**Scenario 5 MscL/K05[5]**	**Scenario 6 MscL/011[6]**	**Scenario 7 MscL/K05[7]**
PHE10	16.42 ± 0.18	16.04 ± 0.22	16.56 ± 0.19	18.11 ± 0.16	17.33 ± 0.46	17.56 ± 0.26	16.50 ± 0.25
ALA11	16.89 ± 0.24	16.36 ± 0.20	17.14 ± 0.30	18.37 ± 0.22	17.84 ± 0.57	17.87 ± 0.29	17.24 ± 0.31
MET12	16.55 ± 0.22	16.02 ± 0.19	16.83 ± 0.40	17.70 ± 0.40	17.86 ± 0.74	16.91 ± 0.31	16.82 ± 0.38
ARG13	13.69 ± 0.20	13.13 ± 0.20	13.89 ± 0.36	14.97 ± 0.43	14.95 ± 0.72	14.15 ± 0.33	13.82 ± 0.39
GLY14	11.96 ± 0.22	11.18 ± 0.23	11.79 ± 0.34	13.05 ± 0.67	12.92 ± 0.74	12.75 ± 0.35	11.63 ± 0.41
**ASN15**	**8.96 ± 0.20**	**8.25 ± 0.20**	**8.69 ± 0.28**	**10.24 ± 0.55**	**10.50 ± 0.81**	**10.15 ± 0.33**	**8.90 ± 0.45**
**VAL16**	**8.57 ± 0.15**	**7.74 ± 0.23**	**8.21 ± 0.18**	**10.51 ± 0.48**	**11.06 ± 0.61**	**10.24 ± 0.26**	**9.85 ± 0.33**
**VAL17**	**10.14 ± 0.12**	**9.64 ± 0.20**	**9.74 ± 0.21**	**12.15 ± 0.36**	**12.15 ± 0.45**	**11.69 ± 0.27**	**11.29 ± 0.33**
**ASP18**	**9.38 ± 0.13**	**9.00 ± 0.17**	**8.96 ± 0.23**	**10.88 ± 0.25**	**11.13 ± 0.66**	**10.34 ± 0.27**	**10.30 ± 0.30**
**LEU19**	**6.84 ± 0.13**	**6.39 ± 0.21**	**6.57 ± 0.20**	**8.36 ± 0.28**	**8.73 ± 0.64**	**7.89 ± 0.26**	**8.02 ± 0.21**
**ALA20**	**7.47 ± 0.12**	**7.24 ± 0.20**	**7.37 ± 0.18**	**9.70 ± 0.22**	**9.50 ± 0.54**	**9.18 ± 0.31**	**9.17 ± 0.21**
**VAL21**	**9.31 ± 0.13**	**9.37 ± 0.13**	**9.12 ± 0.14**	**11.07 ± 0.25**	**11.36 ± 0.56**	**10.54 ± 0.22**	**10.99 ± 0.20**
**GLY22**	**8.19 ± 0.14**	**8.31 ± 0.15**	**8.09 ± 0.14**	**8.94 ± 0.26**	**10.57 ± 0.43**	**8.60 ± 0.19**	**10.35 ± 0.19**
**VAL23**	**6.63 ± 0.13**	**6.56 ± 0.17**	**6.50 ± 0.14**	**7.39 ± 0.13**	**9.10 ± 0.34**	**7.02 ± 0.18**	**9.34 ± 0.17**
**ILE24**	**8.84 ± 0.13**	**8.71 ± 0.15**	**8.63 ± 0.15**	**9.71 ± 0.09**	**10.75 ± 0.32**	**9.38 ± 0.20**	**10.90 ± 0.19**
ILE25	10.86 ± 0.12	10.97 ± 0.15	10.73 ± 0.16	11.04 ± 0.13	12.51 ± 0.33	10.86 ± 0.15	12.60 ± 0.21
GLY26	9.62 ± 0.18	9.90 ± 0.22	9.54 ± 0.25	9.07 ± 0.21	11.13 ± 0.35	9.04 ± 0.22	11.49 ± 0.28
ALA27	8.75 ± 0.16	8.91 ± 0.20	8.64 ± 0.22	8.83 ± 0.20	10.09 ± 0.45	8.65 ± 0.16	10.60 ± 0.30
ALA28	11.56 ± 0.16	11.65 ± 0.18	11.42 ± 0.22	11.83 ± 0.17	12.56 ± 0.40	11.63 ± 0.18	12.85 ± 0.28
PHE29	13.23 ± 0.15	13.44 ± 0.20	13.06 ± 0.24	12.94 ± 0.15	13.99 ± 0.49	12.92 ± 0.14	14.25 ± 0.23
GLY30	11.89 ± 0.17	12.17 ± 0.23	11.65 ± 0.26	11.24 ± 0.20	12.22 ± 0.56	11.33 ± 0.16	12.58 ± 0.24
LYS31	12.19 ± 0.19	12.31 ± 0.21	12.01 ± 0.24	11.72 ± 0.23	12.37 ± 0.43	11.75 ± 0.23	12.57 ± 0.22
ILE32	15.29 ± 0.18	15.40 ± 0.21	15.12 ± 0.24	14.84 ± 0.22	15.38 ± 0.41	14.84 ± 0.23	15.58 ± 0.21
VAL33	15.85 ± 0.15	16.16 ± 0.25	15.80 ± 0.31	15.03 ± 0.24	15.63 ± 0.52	15.14 ± 0.18	16.02 ± 0.20
SER34	14.18 ± 0.18	14.47 ± 0.29	14.01 ± 0.34	12.98 ± 0.20	13.67 ± 0.52	13.22 ± 0.20	13.99 ± 0.19
SER35	15.59 ± 0.26	15.66 ± 0.24	15.17 ± 0.30	13.90 ± 0.16	14.93 ± 0.48	14.07 ± 0.23	15.00 ± 0.18
LEU36	18.42 ± 0.20	18.59 ± 0.25	18.11 ± 0.30	16.44 ± 0.21	17.71 ± 0.45	16.84 ± 0.22	17.85 ± 0.17
VAL37	17.95 ± 0.19	18.22 ± 0.31	17.70 ± 0.32	15.97 ± 0.16	16.82 ± 0.46	16.47 ± 0.21	17.12 ± 0.18
ALA38	16.05 ± 0.23	16.27 ± 0.32	15.73 ± 0.32	14.34 ± 0.24	14.54 ± 0.51	14.68 ± 0.23	14.80 ± 0.24
ASP39	17.48 ± 0.27	17.52 ± 0.29	17.09 ± 0.31	16.27 ± 0.26	16.32 ± 0.54	16.61 ± 0.23	16.40 ± 0.28
ILE40	20.09 ± 0.25	20.04 ± 0.26	19.69 ± 0.30	19.18 ± 0.22	19.45 ± 0.54	19.56 ± 0.23	19.62 ± 0.27

1. MscL only, 1222 snapshots, conventional MD
2. MscL/011A, 1717 snapshots, conventional MD
3. MscL/K05, 1537 snapshots, conventional MD
4. MscL/011A, 10 channel-open conformations collected from the “passing-through” experiment
5. MscL/K05, 23 channel-open conformations collected from the “passing-through” experiment
6. MscL/011A, 204 snapshots, conventional MD from a representative active conformation
7. MscL/K05, 604 snapshots, conventional MD from a representative active conformation
